# The Combination of Two-Dimensional Nanomaterials with Metal Oxide Nanoparticles for Gas Sensors: A Review

**DOI:** 10.3390/nano12060982

**Published:** 2022-03-16

**Authors:** Tao Li, Wen Yin, Shouwu Gao, Yaning Sun, Peilong Xu, Shaohua Wu, Hao Kong, Guozheng Yang, Gang Wei

**Affiliations:** 1 College of Textile & Clothing, Qingdao University, No. 308 Ningxia Road, Qingdao 266071, China; qdlitao2008@126.com (T.L.); yiwin@126.com (W.Y.); qdfzxsyn@163.com (Y.S.); shaohua.wu@qdu.edu.cn (S.W.); 2 State Key Laboratory, Qingdao University, No. 308 Ningxia Road, Qingdao 266071, China; qdgsw@126.com (S.G.); xpl@qdu.edu.cn (P.X.); 3 College of Chemistry and Chemical Engineering, Qingdao University, No. 308 Ningxia Road, Qingdao 266071, China; konghao5@outlook.com (H.K.); yangguozheng123@outlook.com (G.Y.)

**Keywords:** two-dimensional materials, metal oxide, nanoparticles, composite materials, gas sensors

## Abstract

Metal oxide nanoparticles have been widely utilized for the fabrication of functional gas sensors to determine various flammable, explosive, toxic, and harmful gases due to their advantages of low cost, fast response, and high sensitivity. However, metal oxide-based gas sensors reveal the shortcomings of high operating temperature, high power requirement, and low selectivity, which limited their rapid development in the fabrication of high-performance gas sensors. The combination of metal oxides with two-dimensional (2D) nanomaterials to construct a heterostructure can hybridize the advantages of each other and overcome their respective shortcomings, thereby improving the sensing performance of the fabricated gas sensors. In this review, we present recent advances in the fabrication of metal oxide-, 2D nanomaterials-, as well as 2D material/metal oxide composite-based gas sensors with highly sensitive and selective functions. To achieve this aim, we firstly introduce the working principles of various gas sensors, and then discuss the factors that could affect the sensitivity of gas sensors. After that, a lot of cases on the fabrication of gas sensors by using metal oxides, 2D materials, and 2D material/metal oxide composites are demonstrated. Finally, we summarize the current development and discuss potential research directions in this promising topic. We believe in this work is helpful for the readers in multidiscipline research fields like materials science, nanotechnology, chemical engineering, environmental science, and other related aspects.

## 1. Introduction

In actual production and life, flammable, explosive, toxic, and harmful gases pose a serious threat to environmental safety and human health. Therefore, devices with high performance are urgently needed to detect those flammable, explosive, toxic, and harmful gases. The gas sensors play great importance in determining various gases as they can convert a certain gas volume fraction into electrical signals. They have the advantages of low cost, fast response, high sensitivity, and high selectivity. In addition, in some cases, the gas sensor device can be directly used in electronic interfaces. Therefore, gas sensors have been widely used in environmental monitoring, air quality monitoring, vehicle exhaust monitoring, medical diagnosis, food/cosmetics monitoring, and many other fields [1,2,3,4,5,6,7,8].

The fabrication of nanomaterial-based gas sensors has been the focus of research over the past few decades. According to the working principle of the sensors, gas sensors can be divided into several types, such as semiconductor type, polymer type, contact combustion type, and solid electrolyte [9,10,11,12]. Among these gas sensors, the semiconductor gas sensors have developed into one of the largest and most widely used sensors in the world due to their large types of gases, high sensitivity, low price, and simple fabrication [13]. According to the different gas detection methods, semiconductor gas sensors can be divided into two types: resistive and non-resistive, in which the resistive semiconductor gas sensor detects gas concentration according to the change of the resistance value of the semiconductor when it comes into contact with the gas [13]. Currently, gas-sensitive materials such as semiconductor metal oxides, conductive polymers, and carbon materials have been used for the fabrication of resistive semiconductor-type gas sensors [14,15,16,17].

Among these sensing materials for the fabrication of semiconductor gas sensors, metal oxides, including zinc oxide (ZnO), indium oxide (In_2_O_3_), tin oxide (SnO_2_), and tungsten oxide (WO_3_) have been proved to be the best candidates for the fabrication for making resistive gas sensors due to their advantages of simple fabrication, low cost, easy portability, and high sensitivity [18,19,20,21]. However, the high operating temperature, high power, and low selectivity limited their rapid development [22,23]. Two-dimensional (2D) nanomaterials, including graphene, transition metal chalcogenides, layered metal oxides, black phosphorus, and others, have shown great potential in gas sensors due to their unique single-atom-layer structure, high specific surface area, and many surface-active sites [24,25].

2D material-based gas sensors have the advantages of high sensitivity, fast response speed, low energy consumption, and room temperature operation [26,27,28,29,30]. However, since 2D nanomaterials tend to form a dense stack structure during the formation of the conductive network, it is not conducive to the full contact between the flakes inside the conductive network and the gas molecules, making the sensitivity and response recovery speed relatively low at room temperature. Combining metal oxides with 2D nanomaterials to construct a heterostructure can combine the advantages of each other and overcome their respective shortcomings, thereby improving the sensing performance of the fabricated gas sensors. The combination of the metal oxide and 2D materials can drive transformations in the design and performance of 2D nanoelectronics devices, such as the graphene/2D indium oxide/SiC heterostructure [31,32]. Currently, the combination of metal oxides with graphene, transition metal chalcogenide, and other 2D materials to form heterojunction nanostructures for gas sensors have been studied widely, which have exhibited significantly enhanced sensing performance at room temperature [33,34].

In this review, we present the advances in the fabrication and sensing mechanisms of 2D material- and metal oxide nanoparticle-based gas sensors. For this aim, we first introduce the detection mechanism of the resistive semiconductor gas sensors and the factors that can affect the sensitivity of the gas sensors. Then, various types of gas sensors based on metal oxides, 2D materials, and 2D materials/metal oxides composites are introduced. Special emphasis is placed on the recent progress of the combination of metal oxides and 2D nanomaterials for gas sensors. We believe that this review will be helpful for readers to understand the synthesis of functional 2D material-based composites and promote the fabrication of 2D material-based sensors for the high-performance determination of gases.

## 2. Working Principles of Gas Sensors

### 2.1. Mechanism of Oxygen Ion Adsorption on the Surface of Metal Oxide Nanoparticles

**Figure 1:**
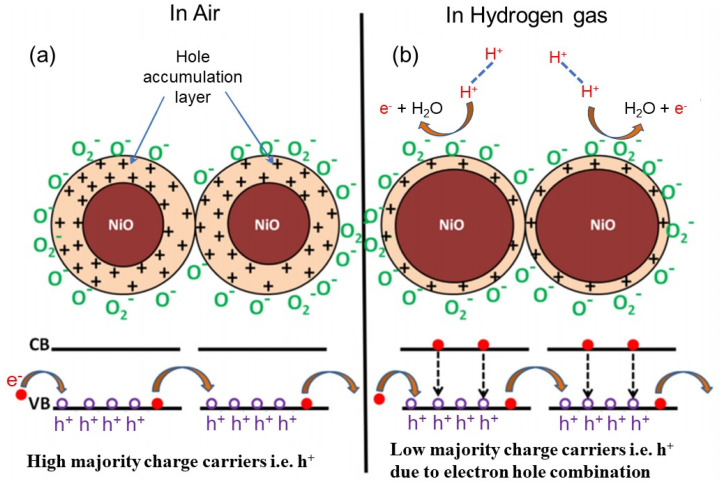
Schematics of H_2_ sensing mechanism for NiO sensor. Hole accumulation of the NiO sensor exposed in air (**a**) and hydrogen (**b**), respectively. Reprinted with permission from Ref. [37]. Copyright 2018 Elsevier.

The sensing mechanism of traditional metal oxide-based gas sensors is related to the resistance change of the sensing materials caused by the adsorption of oxygen ions on the material surface [35,36]. When metal oxides are exposed to air, O_2_ in the air is adsorbed onto the surface of metal oxides, which acts as electron acceptors to extract electrons from the conduction band of oxides and dissociates into different forms of negative oxygen ions (O_2_^−^, O^−^, O^2−^). Since the electrons in the conduction band of the material are captured by oxygen anions, a hole accumulation layer (also called electron depletion layer) rich in hole carriers is formed on the surface of the material, thereby increasing the resistance of the gas-sensing material. Various gases are adsorbed on the surface of metal oxides and interact with oxygen anions to change the electrical conductivity of metal oxides. Taking NiO as an example, when exposed to a reducing gas, the adsorbed oxygen undergoes a redox reaction with the gas, and the captured electrons are released into the conduction band of the semiconductor, and these electrons combine with the holes present in the hole accumulation layer of the sensor material [37]. Therefore, the number of carriers is reduced, and the resistance value is increased, so as to achieve the purpose of gas detection, as shown in Figure 1.

### 2.2. Charge Transfer Mechanism of 2D Material-Based Gas Sensors

**Figure 2:**
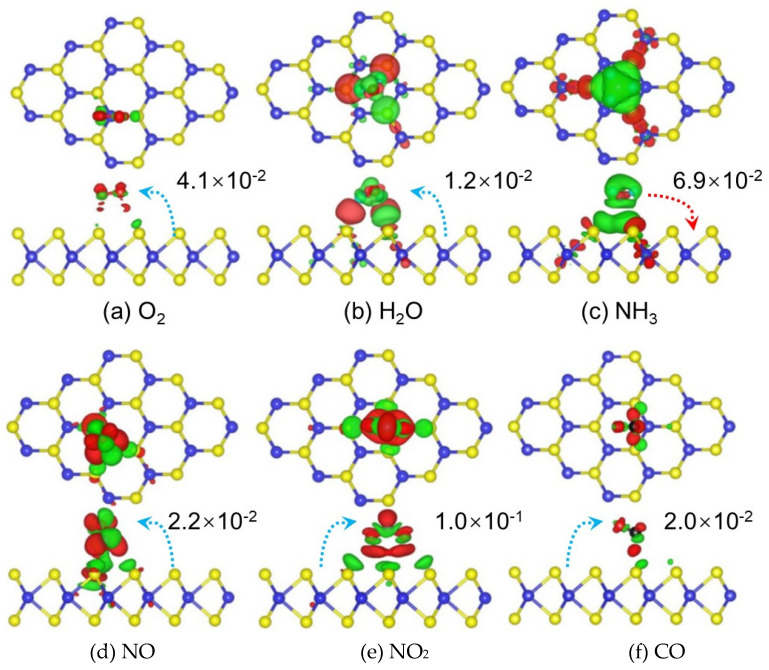
Charge density difference plots for (**a**) O_2_, (**b**) H_2_O, (**c**) NH_3_, (**d**) NO, (**e**) NO_2_, and (**f**) CO interacting with monolayer MoS_2_. Reprinted with permission from Ref. [39]. Copyright 2013 Springer.

The sensing mechanism of gas sensors based on graphene and related 2D layered materials is mainly related to the charge transfer process, in which the sensing material acts as a charge acceptor or donor [38]. When exposed to different gases, a charge transfer reaction occurs between the sensing material and the adsorbed gas, and the direction and amount of charge transfer are different, resulting in different changes in the material resistance. Taking layered MoS_2_ as an example, the charge transfer between different gas molecules (including O_2_, H_2_O, NH_3_, NO, NO_2_, CO) and monolayer MoS_2_ is different [39], as shown in Figure 2.

Before gas adsorption, some electrons already exist in the conduction band of the n-type MoS_2_ monolayer. When MoS_2_ is exposed to O_2_, H_2_O, NO, NO_2_, and CO gases, the electron charge is transferred from MoS_2_ to the gas atmosphere, resulting in a decrease in the carrier density of MoS_2_ and an increase in the resistance of MoS_2_. On the contrary, When MoS_2_ is exposed to NH_3_, the NH_3_ molecules adsorbed on MoS_2_ act as charge donors to transfer electrons to the MoS_2_ monolayer, increasing the carrier density of the MoS_2_ monolayer and reducing its resistance (Figure 2).

### 2.3. Gas Sensing Mechanism of 2D Material/Metal Oxide Composites

**Figure 3:**
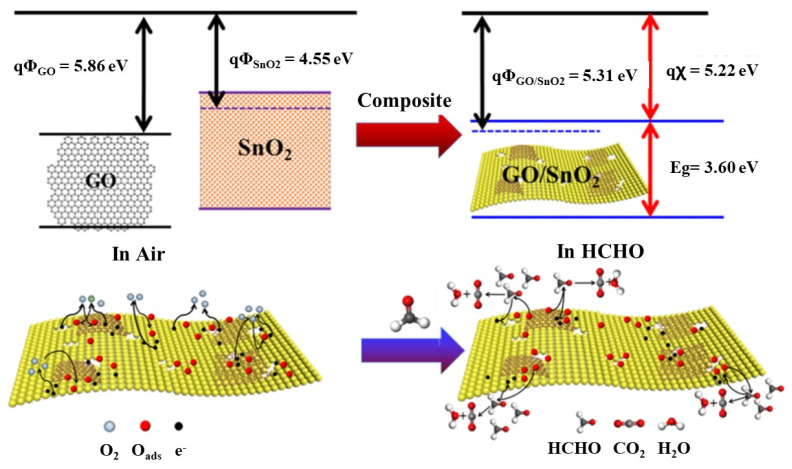
Schematic diagram of the sensing mechanism for GO/SnO_2_. Reprinted with permission from Ref. [40]. Copyright 2019 ACS.

When one material is composited with another, the bonding between different materials forms p-n, n-n, p-p, and Schottky heterojunctions. Among them, the formation of p-n heterojunction is beneficial for adjusting the thickness of the electron depletion layer, thereby further improving the sensing performance of the fabricated gas sensors. For example, when SnO_2_ is combined with graphene oxide (GO), the p-n heterojunction can be formed to form a new energy-level structure, as shown in Figure 3 [40]. The electron dissipation layer expands at the interface of SnO_2_ and GO, resulting in increased resistance. When formaldehyde is introduced, the trapped electrons are released back into the conduction band, resulting in a reduction in the width of the dissipation layer, which reduces the resistance of the sample. The porous and ultrathin structure of the SnO_2_/GO composite increases the specific surface area and active sites, facilitates the reaction with HCHO gas, and contributes to the ultrahigh response for gas sensing. It should be noted that the ultrathin nanosheet structure of GO shortens the transport path and greatly improves the response of the gas sensor. Meanwhile, the abundant pores in SnO_2_ are favorable for gas diffusion and help to improve the response/recovery performance. In addition, GO can act as a spacer, which reduced the agglomeration of SnO_2_ nanoparticles, and provided abundant adsorption sites for HCHO gas, thereby enhancing the gas sensing response.

## 3. Factors for Affecting the Sensitivity of Gas Sensors

### 3.1. Size, Morphology, and Porosity

**Figure 4:**
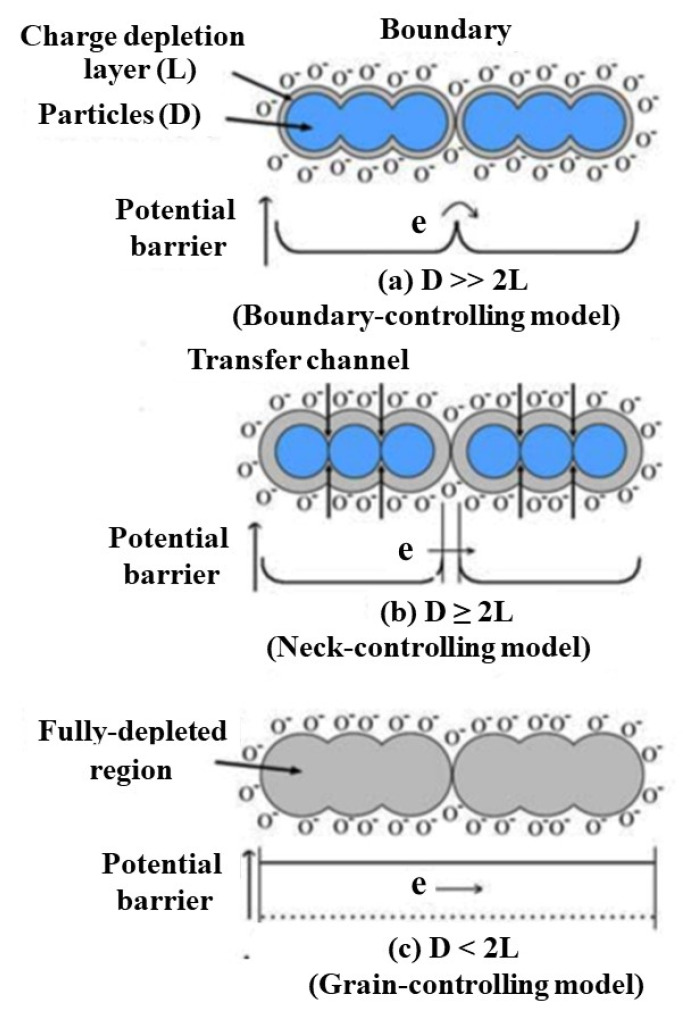
Schematic model of the effect of the crystallite size on the sensitivity of metal-oxide gas sensors: (**a**) D >> 2L, (**b**) D ≥ 2L, and (**c**) D < 2L. Reprinted with permission from Ref. [42]. Copyright 1991 Elsevier.

The grain size, morphology, and porosity are important factors for affecting the sensing performance of semiconductor-based gas sensors. In gas sensors, semiconductor nanoparticles are connected to adjacent particles through grain boundaries to form larger aggregates [41]. On the particle surface, the adsorbed oxygen molecules extract electrons from the conduction band and capture electrons in the form of ions on the surface, resulting in band bending and electron depletion layers, as shown in Figure 4 [42,43]. Since the transport of electrons between grains needs to pass through the electron depletion layer, the grain size has a great influence on the conductivity, which in turn affects the gas sensing performance of the material-based gas sensor. When the particle size (D) is much larger than twice the thickness (L) of the electron depletion layer (D >> 2L), there is a wide electron channel between the grains, and the gas sensitivity of the material is mainly controlled by the surface of the nanoparticles (boundary control). When D ≥ 2L, there is a constricted conduction channel. The change in electrical conductivity depends not only on the particle boundary barrier but also on the cross-sectional area of the channel, and the gas sensitivity of the material is mainly controlled by the contact neck between nanoparticles (neck control). When D < 2L, the electron depletion layer dominates, the entire nanoparticle is contained in the electron depletion layer (grain control), and the sensitivity of the material is very high. The energy bands are nearly flat throughout the interconnected grain structure, and there is no significant impediment to the inter-grain charge transport. The small amount of charge gained from the surface reaction results in a large change in the conductivity of the entire structure. Therefore, the smaller grain size is beneficial to improving the sensitivity of the gas sensor. When the grain size is small enough, the crystal becomes very sensitive to the surrounding gas molecules. For example, Min et al. used SnO_2_ as the sensing material to prepare SnO2 films with different particle sizes (8.4–18.5 nm) and different porosity (70.8–99.5%), and found that in the gas sensors with porous structure, high porosity and low average particle size exhibited quicker gas sensing response [44].

However, when the grain size is excessively reduced, the agglomeration between particles is serious. If the aggregates are relatively dense, only the particles on the surface of the aggregates could participate in the gas sensing reaction, and the internal materials are wasted because they are not in contact with the gas, resulting in a decrease in the utilization rate of the material. In addition, the agglomeration is not conducive to the diffusion of gas inside the material and will reduce the gas sensing performance [45,46]. Increasing the specific surface area not only facilitates the adsorption of oxygen molecules in the air on the surface of the material, but also increases more effective active sites and more gas transmission channels to facilitate the diffusion and absorption of the test gas. Therefore, increasing the specific surface area of gas-sensing materials is an important way to modify the sensing properties of gas sensors. The regulation of both the morphology (flower-like, sea urchin-like, etc.) and porous structure (macropores, mesopores, and micropores) of materials is an effective way to improve the specific surface area of materials. Nanomaterials with porous structures can increase the effective surface area and active sites of the material due to their special pore structure, so that the material has better permeability, making gas molecules easier to diffuse into the interior of the material, and increasing the contact between the material and the gas. It can accelerate the diffusion of gas, improve the response and recovery speed of the gas sensor, and thus improve its gas sensing performance.

For example, Boudiba et al. synthesized WO_3_ materials with different morphologies by direct precipitation, ion exchange, and hydrothermal methods, and further used the as-prepared materials to fabricate gas sensors. Their results indicated that the greater the porosity, the higher the sensitivity to SO_2_ gas [47]. In another case, Jia et al. prepared WO_3_ semiconductor materials with different morphologies by hydrothermal method as sensitive materials. Under the same test conditions, they found that the sensitivity of WO_3_ nanorods towards acetone was 19.52, while the sensitivity of WO_3_ nanospheres towards acetone was 25.71. In addition, WO_3_ nanoshpheres exhibited better selectivity than nanorods [48]. Lü et al. [49] successfully prepared porous materials with extremely high specific surface area (120.9 m^2^·g^−1^) by simple chemical transformation of Co-based metal-organic frameworks (Co-MOFs) template and controlling the appropriate calcination temperature (300 °C). The prepared Co_3_O_4_ concave nanocubes were systematically tested for their gas-sensing properties to volatile organic compounds (VOCs), including ethanol, acetone, toluene, and benzene. to the fabricated sensors exhibited excellent performance in gas sensing, such as high sensitivity, low detection limit (10 ppm), fast response and recovery (<10 s), and high selectivity for ethanol. Wang et al. synthesized concave Cu_2_O octahedral nanoparticles with a diameter of about 400 nm and performed gas-sensing tests for benzene (C_6_H_6_) and NO_2_ [50]. It was found that the concave Cu_2_O octahedral nanoparticles exhibited better gas sensing properties than Cu_2_O nanorods. Unlike conventional octahedrons, Cu_2_O octahedral nanoparticles have a structure similar to icosahedral with sharp boundaries. Therefore, compared with nanorods, the synthesized Cu_2_O octahedral nanoparticles have a larger specific surface area, which can provide more reactive sites and thus exhibit better gas sensing properties.

### 3.2. Doping of Metals

The doping of metal elements can effectively improve the gas recognition ability of gas sensing materials, and is an important method to improve gas-sensing performance. Different dopant species may lead to different types of crystallites, defects and electronic properties [51,52]. Doping or surface modification by adding metal elements (such as Ag, Au, Pt, Pd, etc.) on the surface of the gas-sensing materials can increase the number of active sites, promote the adsorption/desorption reaction on the surface of the gas-sensing materials, and reduce the reaction activation energy, and reduce the operating temperature, thereby improving the gas-sensing performance [53].

For instance, Fedorenko et al. [54] prepared Pd-doped SnO_2_ semiconductor sensors by a sol–gel method. The effect of Pd additives on methane sensitivity was studied, and it was found that due to the catalytic activity of Pd, compared with undoped materials, the addition of Pd to SnO_2_ significantly improved the sensor response to methane (about 6–7 times). Barbosa et al. [55] studied the sensing responses of SnO micro-sheets that modified with Ag and Pd noble metal catalysts towards the gases such as NO_2_, H_2_, and CO, and found that the Ag/Pd surface-modified SnO micro-sheets exhibited higher sensitivity to gases such as H_2_ and CO. However, the catalyst particles reduced the sensing response to oxidizing gases such as NO_2_. It is clear that the catalytic activity of Pd nanoparticles is related to chemical sensitization, while the catalytic activity of Ag nanoparticles is related to electronic sensitization. The Ag-modified samples showed high response to H_2_, and Pd-modified samples showed high response and selectivity to CO. Zhang et al. synthesized Co-doped sponge-like In_2_O_3_ cubes by a simple and environmentally friendly hydrothermal method with the help of organic solvents, and studied their acetone gas-sensing properties [56]. It was found that Co-doped In_2_O_3_ has good gas-sensing performance for acetone gas, and its porous structure can create more adsorption sites for the adsorption of oxygen molecules and the diffusion of the target gas, thereby significantly improving the sensing performance. Compared with the undoped sample, the response value of the doped Co-In_2_O_3_ sample to acetone was increased by 3.25 times, the response recovery time was fast (1.143 s/37.5 s), the detection limit was low (5 ppm), the reproducibility was good, and the selectivity was high. In another case, Ma et al. reported a Pt-modified WO_3_ mesoporous material with high sensitivity to CO [57]. Pt acted as a chemical sensitizer, and gas molecules were adsorbed and flowed into the gas-sensitive material through the spillover effect. In addition, the PtO formed on the surface of Pt further increased the electron depletion layer, and enhanced the electron sensitivity. The synergistic effects of both components further improved the gas-sensing performance.

Compound doping is another important method to improve the sensing performance of gas sensors. When a metal oxide is combined with other metal oxides, a heterojunction structure is constructed. Since the two materials each have their own Fermi energy levels, there will be a mutual transfer of carriers between the two materials to form a space charge layer, so as to achieve the purpose of enhancing the gas-sensing properties of the compound materials. For example, Ju et al. [58] prepared SnO_2_ hollow spheres by a template-assisted hydrothermal method and successfully implanted p-type NiO nanoparticles onto the surface of SnO_2_ hollow spheres by the pulsed laser deposition (PLD) to prepare NiO/SnO_2_ p-n hollow spheres. The gas-sensing performance test indicated that its response to 10 ppm triethylamine (TEA) gas could reach 48.6, which was much higher than that of the original SnO_2_ hollow spheres, and the detection limit was as low as 2 ppm. The optimal operating temperature dropped to 220 °C, which was 40 °C lower than that of the original SnO_2_ hollow sphere sensor. Compared with the pristine SnO_2_ sensor, the enhanced response of NiO/SnO_2_ sensor to TEA is mainly attributed to the formation of a depletion layer by the p-n heterojunction interface, which makes the resistance of hybrid materials in air and TEA gas change a lot.

In addition, when the gas-sensing materials of different dimensions are compounded, the stacking of the gas-sensing materials can be prevented, the porosity can be increased, and the gas-sensing performance can be improved. For example, Kida et al. [59] introduced monodispersed SnO_2_ nanoparticles (about 4 nm) into WO_3_ nanosheet-based films, which could improve its porosity, prevent the aggregation of flakes, and increase the diffusion paths and adsorption sites of gas molecules. The response sensitivity of the composites to NO_2_ was enhanced when the concentration in air was 20–1000 ppb, indicating the effectiveness of the microstructure control of the WO_3_-based film on high-sensitivity NO_2_ detection. In another case, Mishra et al. [60] prepared nanocubic In_2_O_3_@RGO composites by combining In_2_O_3_ with reduced graphene oxide (RGO), and the sensor based on nanocubic In_2_O_3_@RGO heterostructures exhibited high resistance to acetone (~85%) and formaldehyde (~88%) with good selectivity, long-term stability, and fast response/recovery rates

## 4. 2D Material-Based Gas Sensors

With the successful preparation of graphene materials, its unique structure and excellent properties have attracted widespread attention, thus setting off a research upsurge in 2D materials. 2D nanomaterials have a large specific surface area and special electrical properties due to their nanoscale thin-layer structure. After the gas is adsorbed on the surface, it will affect the conductivity of the surface, so it can be used as a gas-sensing material to adsorb and capture certain single species of gas molecules, with excellent gas-sensing properties. Gas sensors based on 2D nanomaterials exhibit many advantages, such as high sensitivity, fast response speed, low energy consumption, and the ability to work at room temperature.

### 4.1. 2D Graphene-Based Gas Sensors

Graphene is a honeycomb 2D carbon nanomaterial composed of a single layer of sp^2^ carbon atoms. It is currently the thinnest 2D material in the world, with a thickness of only 0.35 nm. Graphene has many excellent properties due to its special structure, such as good electrical conductivity, high carrier mobility, transparency, and mechanical strength. As a typical 2D material, every atom in the graphene structure can be considered as a surface atom, so ideally every atom can interact with the gas, which makes graphene promising as a kind of gas sensor with ultrahigh sensitivity. In the process of adsorption and desorption of gas molecules, graphene nanosheets will affect the change of local carrier concentration in the material, thus showing the transition of electrical signal in the detection of electrochemical performance, and has a good development prospect in gas adsorption. In 2007, Novoselov’s group first reported a graphene-based gas sensor, which confirmed that a graphene-based nanoscale gas sensor can be used to detect the adsorption or desorption of single gas molecules on the graphene surface [61]. This study opens the door to research on 2D graphene-based gas sensors.

**Figure 5:**
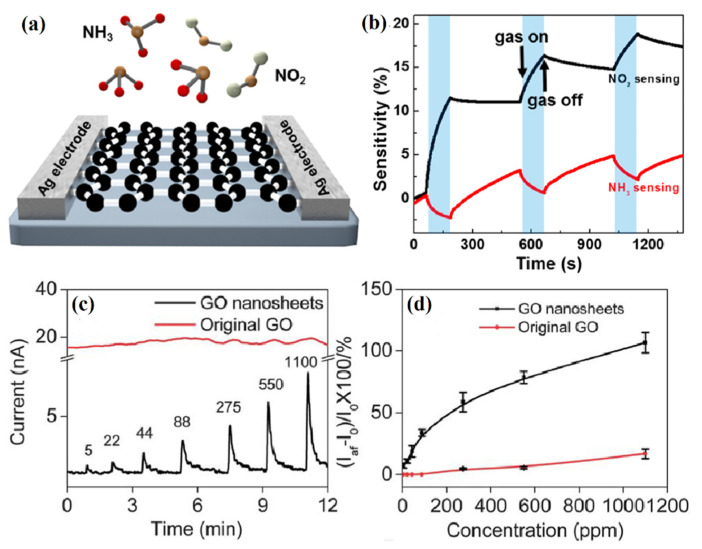
Schematic (**a**) and time-resolved sensitivity (**b**) of the graphene sensor toward NH_3_ and NO_2_ gas molecules. (**c**) Current vs. time curves for 5–1100 ppm of SO_2_ for the original GO and edge-tailored GO nanosheets, and (**d**) the corresponding sensitivities of the sensors to SO_2_ gas. Reprinted with permission from Ref. [69]. Copyright 2016 ACS.

Single-layer graphene nanosheets, RGO, chemically modified graphene, and GO have been proven to be good gas sensing materials [62,63,64]. Since the main advantage of graphene nanostructures is the low temperature response, this sensor can greatly reduce the energy consumption of the sensing device. Various graphene-based gas sensors have been used to detect various harmful gases such as NO_2_, NH_3_, CO_2_, SO_2_, and H_2_S. For example, Ricciardella et al. developed a graphene film-based room temperature gas sensor with a sensitivity of up to 50 ppb (parts-per-billion) to NO_2_ [65]. Various methods, such as mechanical exfoliation, chemical vapor deposition (CVD), and epitaxy, have been used to prepare graphene for gas sensing applications. For example, Balandin et al. [66] prepared monolayer graphene using a mechanical exfoliation method and reported a monolayer intrinsic graphene transistor, which can utilize low-frequency noise in combination with other sensing parameters to realize selective gas sensing of monolithic graphene transistors. Choi et al. [67] prepared graphene by CVD and transferred it onto flexible substrates, and demonstrated a gas sensor using graphene as a sensing material on a transparent (Tr > 90%) flexible substrate. Nomani et al. demonstrated that epitaxial growth of graphene on Si and C surfaces of semi-insulating 6H-SiC substrates can provide very high NO_2_ detection sensitivity and selectivity, as well as fast response times [68]. Yang et al. directly grew multilayer graphene on various substrates through the thermal annealing process of catalytic metal encapsulation, and tested it as a gas sensor for NO_2_ and NH_3_ gas molecules to detect its response sensitivity to NO_2_ and NH_3_ [69]. The schematic diagram of the graphene sensor is shown in Figure 5a. The NO_2_ molecules are electron acceptors (p-type dopant), which extract electrons from graphene, while the NH_3_ molecules are electron donors (n-type dopant), which donate electrons to graphene. Therefore, when NO_2_ molecules are adsorbed, the conductivity of graphene is enhanced, while when NH_3_ molecules are adsorbed on the graphene surface, the conductivity decreases due to the compensation effect (Figure 5b).

GO is suitable for gas sensors due to its multiple properties, such as easy processing, high solubility in various solvents, and containing oxygen functional groups or defects. Since the defects or functional groups in GO can act as reaction sites for gas adsorption, making the gas easily adsorbed on the surface of GO and improving the selectivity and sensitivity of the GO-based sensor, the response of the GO-based sensor can be tuned by functionalization. Shen et al. [70] prepared edge-trimmed GO nanosheets by periodically acid-treating GO, and then fabricated field effect transistors (FETs) for gas sensing testing of SO_2_ at room temperature (Figure 5c,d). Compared with pristine GO nanosheets, edge-clipped GO nanosheets were found to have a significant response enhancement effect to SO_2_ gas, and the detection concentration range was 5–1100 ppm. Meanwhile, the edge-trimmed GO device also exhibited a fast response time, which was mainly attributed to the hygroscopic properties of the GO nanosheets, which can trap water molecules and react with SO_2_ to generate sulfuric acid to facilitate their fast protonation process. By utilizing different reducing agents to remove oxygen from GO and recover aromatic double-bonded carbons, the selectivity of RGO-based gas sensors could be improved significantly. For example, Guha et al. [71] developed a gas sensor for NaBH_4_ reduction of GO on a ceramic substrate and reported its performance for detecting NH_3_ at room temperature. The response to NH_3_ can be optimized by the reduction time of GO. Through chemical modification, RGO can introduce some foreign groups or atoms to change its surface properties, which can enhance its sensing performance. For example, the response of RGO reduced with p-phenylenediamine (PPD) to dimethyl methylphosphonate (DMMP) was 4.7 times higher than that of RGO reduced by ordinary methods [72]. The RGO-based gas sensor reduced by ascorbic acid has high selectivity for corrosive NO_2_ and Cl_2_, and the detection limit can reach 100 and 500 ppb, respectively [73].

### 4.2. 2D Transition Metal Sulfide-Based Gas Sensors

**Figure 6:**
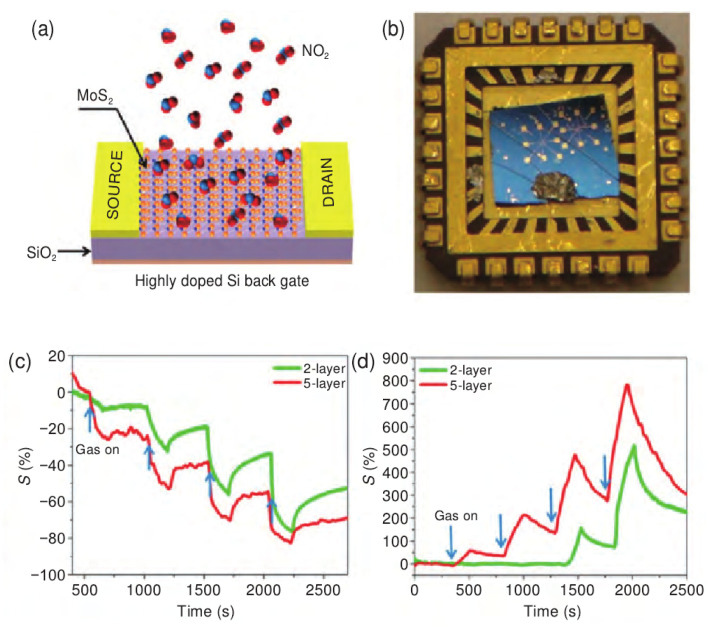
Sensing behavior of atomically thin-layered MoS_2_ transistors. (**a**) Schematic of the MoS_2_ transistor-based NO_2_ gas-sensing device. (**b**) Optical photograph of the MoS_2_ sensing device mounted on the chip. Comparative two- and five-layer MoS_2_ cyclic sensing performances with NH_3_. (**c**) and NO_2_ (for 100, 200, 500, 1000 ppm) (**d**). Reprinted with permission from Ref. [78]. Copyright 2013 ACS.

As a typical p-type inorganic 2D material with a hexagonal filled layered structure of TMDs, it has received extensive attention in energy conversion and storage, especially in room temperature gas sensors, which have unique advantages and are widely used in various gas detection. Similar to graphene, MoS_2_ consists of vertically stacked layers, each formed by covalently bonded Mo-S atoms, with adjacent layers connected by relatively weak van der Waals forces. The weak van der Waals interactions allow gas molecules to permeate and diffuse freely between the layers, so the resistance of MoS_2_ can change dramatically with the adsorption and diffusion of gas molecules within the layers. Various methods for gas sensing using few-layer MoS_2_ have been reported in the literature, including detectors for many kinds of chemical vapors such as H_2_, NO_2_, and ethanol [74,75,76]. For example, Li et al. [77] found for the first time that mechanically exfoliated multilayer MoS_2_ exhibited high sensitivity to NO gas, while monolayer MoS_2_ had an unstable response to NO gas. They used a mechanical lift-off technique to deposit monolayer and multilayer MoS_2_ films on Si/SiO_2_ surfaces for the fabrication of FETs. The FET acted as a gas sensor, which realized gas detection by monitoring the change of the conductance of the FET channel during the adsorption of target gas molecules. Since the mechanically cut MoS_2_ sheet is an n-type semiconductor, when the MoS_2_ channel was exposed to NO gas, it would result in p-doping of the channel, resulting in an increase in channel resistance and a decrease in current flow. It was found that although the single-layer MoS_2_ device exhibited a fast response after exposure to NO, the current was unstable; the two-layered, three-layered, and four-layered MoS_2_ devices all exhibited stable and sensitive responses to NO at a concentration of 0.8 ppm. Late et al. [78] systematically studied the relationship between the number of MoS_2_ layers and gas sensing performance, and found that the sensitivity and recovery time of 5-layered MoS_2_ to NH_3_ and NO_2_ gases were better than those of double-layered MoS_2_ (Figure 6a–d). These findings suggest that a small amount of layered MoS_2_ has great potential to detect various polar gas molecules.

**Figure 7:**
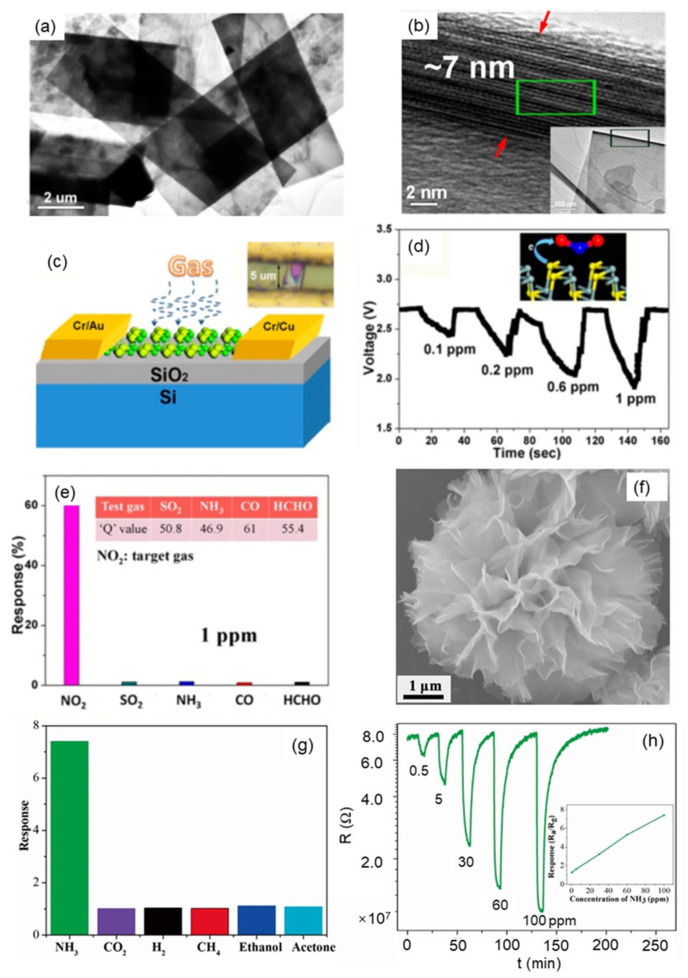
TEM (**a**) and HRTEM (**b**) images of 2D thin SnS crystals. Inset in (**b**) is the corresponding low-magnification TEM image. (**c**) Schematic structure of SnS thin-crystal-based gas-sensor device. The inset shows the optical image of the device. (**d**) Real-time voltage response after exposure of the device to NO_2_ gas with increased concentration. The inset schematically illustrates the electron transfer process from SnS to NO_2_. (**e**) Selectivity of the sensor to a series of gases of 1 ppm. Inset shows the Q values of the SnS thin crystal sensor for NO_2_ as a target gas. Reprinted with permission from Ref. [79]. Copyright 2016 ACS. (**f**) Representative FESEM image of the flower-like SnS_2_ synthesized by a facile solvothermal technique. (**g**) Sensor responses of the SnS_2_ based sensor upon exposure to six kinds of gases at 200 °C. (**h**) Typical response-recovery characteristic of the SnS_2_ based sensor to different concentrations of NH_3_ gas at 200 °C (Inset shows the corresponding response curve). Reprinted with permission from Ref. [80]. Copyright 2018 Elsevier.

At present, 2D Sn-based sulfide materials (SnS and SnS_2_) are also used in the field of gas sensors due to their unique performance advantages. For example, Wang et al. [79] successfully synthesized free-standing large-scale ultrathin SnS crystalline materials by utilizing the 2D directional attachment growth of colloidal quantum dots in a high-pressure solvothermal reaction. The SnS ultrathin crystals were rectangular with uniform shape, the lateral dimension was between 20 and 30 μm, and the thickness was less than 10 nm (Figure 7a,b). The obtained material was used to fabricate a gas sensor, which exhibited excellent sensitivity and selectivity for NO_2_ at room temperature with a detection limit of 100 ppb (Figure 7c–e). Xiong et al. [80] synthesized 3D flower-like SnS_2_ nanomaterials assembled from nanosheets and fabricated them into gas sensors by a simple solvothermal method, As shown in Figure 7f–h. When 100 ppm NH_3_ was detected at 200 °C, the response value was 7.4, the response time was 40.6 s, and the recovery time was 624 s. The prepared nanoflowers have good selectivity to NH_3_ with a detection limit of 0.5 ppm. This study attributes the excellent performance of the SnS_2_ sensor for NH_3_ to the unique thin-layer flower-like nanostructure, which is beneficial to the carrier transfer process and gas adsorption/desorption process [23].

In order to further improve the gas sensing performance of TMD materials, people have improved the gas-sensing performance through external energy strategies (ultraviolet-assisted irradiation or applying a bias voltage, etc.) or composite strategies with other materials. For example, Late et al. found that 5-layer MoS_2_ has a more sensitive response to both NH_3_ and NO_2_ than 2-layer MoS_2_ with the assistance of a bias voltage (+15 V). The photoconverted radiation of 4 mW/cm^2^ can increase the sensitivity of NO_2_ gas sensing, while the light intensity of 15 mW/cm^2^ can reduce the recovery time [78]. Wu et al. [81] prepared MoTe_2_ nanosheets by mechanical exfoliation, and the sensitivity to NO_2_ under 254 nm UV light was significantly improved by one order of magnitude compared with the dark condition, and the detection limit was significantly reduced to 252 ppt. Gu et al. [82] synthesized 2D SnS_2_ nanosheets by a solvothermal method, and after irradiation with a 520 nm green LED lamp, realized NO_2_ detection at room temperature, with good repeatability and selectivity for 8 ppm NO_2_ in a dry environment and the response value was 10.8. Cheng et al. [83] combined the excellent sensing performance and gas adsorption capacity of 2D SnS_2_ hexagonal nanosheets with the good electrical properties of graphene, and used the excellent electrical conductivity of graphene to make up for the shortcoming of the poor conductivity of SnS_2_ at room temperature and prepared a high-performance RGO/SnS_2_ heterojunction-based NO_2_ sensor. Compared with the single SnS_2_ gas sensor, the graphene-doped sensor exhibited better selectivity to NO_2_, while effectively reducing the optimal operating temperature of the device, and its response to 5 ppm NO_2_ gas increased by nearly one order of magnitude, and the response recovery time was reduced to less than a minute. Compared with the single SnS_2_ gas sensor, the graphene-doped sensor exhibited good selectivity to NO_2_, while effectively reducing the optimal operating temperature of the device, and its response to 5 ppm NO_2_ gas increased by nearly one order of magnitude, and the response recovery time was shortened to less than one minute.

### 4.3. 2D Metal Oxide Based Gas Sensors

2D semiconductor oxide nanosheets are also commonly used 2D materials in the field of gas sensors. Among them, layered MoO_3_, WO_3_ and SnO_2_ have attracted much attention due to their stability in high-temperature air [84,85,86]. For example, Cho et al. [87] reported the preparation of MoO_3_ nanosheets by ultrasonic spray pyrolysis, and studied their gas-sensing properties, and found that there was still a response even when the gas concentration of trimethylamine was lower than 45 ppb. The super sensitivity to trimethylamine gas is inseparable from its larger specific surface area, as ultrathin nanosheets with the larger specific surface area can provide a larger electron depletion layer and a faster gas diffusion rate across the nanosheets. In addition, MoO_3_ is an acidic oxide, which is more likely to react with basic gas preferentially, so it has super selective properties for basic gas trimethylamine. Wang et al. [88] prepared WO_3_ porous nanosheet arrays by chemical bath deposition, and found that WO_3_ arrays composed of 20 nm ultrathin porous nanosheets had better low-temperature NO_2_ gas sensing properties. At an operating temperature of 100 °C, the response to 10 ppm NO_2_ was as high as 460.

For 2D metal oxide nanomaterials, the difference in exposed crystal planes will affect their gas sensing properties. For example, Kaneti et al. used a simple and effective hydrothermal method to prepare ZnO nanosheets. By simulating the adsorption of gas molecules on the surfaces of different ZnO crystals, it was found that the enhanced gas-sensing performance of ZnO nanosheets is related to the exposed surface, the (10$\bar{1}$0) face of ZnO possesses better adsorption capacity for n-butanol than the (11$\bar{2}$0) face and (0001) face, showing higher responsiveness, better selectivity, and higher stability [89]. In addition, Wang et al. [90] used a two-step method to synthesize ultrathin porous In_2_O_3_ nanosheets with uniform mesopores and found that they exhibited an ultra-high response to 10 ppb NOx at a lower operating temperature (120 °C), its sensitivity response value was 213, and the response time was 4 s. The thickness of the ultrathin nanosheets is about 3.7 nm, with a large number of active reaction sites, which can enhance the response to NOx, and the porous structure can shorten the gas transmission path and enhance the gas diffusion efficiency, thereby improving the gas sensing performance. Wang et al. synthesized Co_3_O_4_ mesh nanosheet arrays for the detection of NH_3_ [91]. The porous mesh structure promoted gas diffusion and provided a larger active reactive surface to react with the target gas, thereby improving the gas sensing performance. Therefore, even when the NH_3_ concentration is 0.2 ppm, the sensor still has obvious response characteristics. The response/recovery time of Co_3_O_4_ nanosheet arrays to 0.2 ppm NH_3_ is 9 s/134 s, showing good reproducibility and long-term room temperature stability.

2D nanostructures have shown great potential in the field of gas sensing due to their high specific surface area and highly efficient active sites on exposed surfaces. To further enhance the gas-sensing properties of 2D metal oxides, ion doping or surface modification on them is a valuable approach to enhance the response and recovery properties. For example, Chen et al. [92] prepared 2D Cd-doped porous Co_3_O_4_ nanosheets by microwave-assisted solvothermal method and in situ annealing process, and investigated their sensing performance for NO_2_ at room temperature. It was found that 5% Cd-doped Co_3_O_4_ nanosheets significantly improved the response to NO_2_ at room temperature (3.38), decreased the recovery time (620 s), and lowered the detection limit to 154 ppb. The reason for the performance improvement is that Cd doping mainly promotes the adsorption of NO_2_ through a series of factors such as enhancing the electronic conductivity, increasing the concentration of oxygen vacancies, and forming Co^2+^ - O^2−^, thus promoting its excellent room temperature sensing performance.

### 4.4. Other 2D Material-Based Gas Sensors

MXene is a new type of 2D material with layered structure discovered in recent years, which is generally transition metal carbide or carbon-nitrogen compound, and is a MAX ternary phase material. Its general structural formula is M_n+1_AX_n_ (n = 1, 2 or 3), where M is one of transition metal elements, A is one of the main group elements (mainly III, IV group elements), X It is carbon or nitrogen, and there are more than 70 kinds of MAX materials. MXene materials are 2D materials formed by extracting element A in MAX. The general formula is M_n+1_X_n_ (n = 1, 2 or 3) [93,94]. Due to the characteristics of conventional semiconductor materials and the fact that a large number of functional groups and other active sites remain on the surface after etching, which facilitates subsequent modification, such materials have great application potential in the field of sensing [94,95].

Xiao et al. applied MXene nanomaterials to the detection of NH_3_ gas in 2015 [83]. Since the successful synthesis of 2D compound MXenes by Gogotsi et al. in 2011, the application of 2D MXene nanomaterials in gas sensing has been continuously developed [96,97,98]. For example, Lee et al. [99] reported a Ti_3_C_2_T_x_-based gas sensor. After studying the room-temperature gas sensing performance of Ti_3_C_2_T_x_ nanosheets on flexible polyimide, it was found that the Ti_3_C_2_T_x_ sensor exhibited p-type sensing behavior for reduced gases, with a theoretical detection limit of 9.27 ppm for acetone. Based on the charge interaction between gas molecules and Ti_3_C_2_T_x_ surface functional groups -O and -OH, the sensing mechanism of Ti_3_C_2_T_x_ is proposed. Chae et al. [100] investigated the dominant factors affecting the oxidation rate of Ti_3_C_2_T_x_ flakes and their corresponding sensing properties. In order to improve the sensing performance of MXenes, the gas sensing performance of MXene-based sensors has been realized by surface chemistry and composite structure. Yang et al. [101] prepared organic-like Ti_3_C_2_T_x_ by HF acid etching, added the prepared powder to NaOH solution, and used alkali treatment to demonstrate the effect of surface groups on its sensing performance. It was found that the response of the alkali-treated Ti_3_C_2_T_x_ sensor to 100 ppm NH_3_ at room temperature was two times higher than that of the untreated one. This is due to the adsorption of N atoms in NH_3_ molecules on top of Ti atoms in Ti_3_C_2_T_x_ to form strong N-Ti bonds. Alkaline treatment increased the -O end, increased N-Ti bond, and promoted the increase of NH_3_ adsorption. Furthermore, after oxygen functionalization, Ti_3_C_2_T_x_ increased the resistance by transitioning to a semiconductor, thereby increasing the gas response signal. Besides Ti_3_C_2_T_x_, 2D MXenes such as V_2_CT_x_ and Mo_2_C have also been investigated for gas sensors [102,103]. The 2D V_2_CT_x_ sensor composed of monolayer or multilayer 2D V_2_CT_x_ on polyimide film fabricated by Lee et al. [102] can measure polar gases (hydrogen sulfide, ammonia, acetone, and ethanol) and non-polar gases at room temperature (hydrogen and methane). The V_2_CT_x_ sensor shows ultrahigh sensitivity for non-polar gases, with minimum detection limits of 2 ppm and 25 ppm for hydrogen and methane, respectively.

## 5. Metal Oxide Nanomaterials-Based Gas Sensors

Because of its large specific surface area, high surface activity, many active sites and sensitive to the surrounding environment, the gas sensor prepared by metal oxide nanomaterials has high response sensitivity and fast response-recovery speed. According to the semiconductor type, metal oxide semiconductors can be divided into n-type and p-type. In n-type semiconductors, including SnO_2_, ZnO, TiO_2_, In2O_3_, etc., the carriers are mainly free electrons. However, in p-type semiconductors, such as CuO, NiO, Co_3_O_4_, etc., the carriers are mainly holes. When n-type semiconductors are exposed to reducing gases (such as ethanol, NH_3_, H_2_, etc.), the resistance of the materials will decrease, while when exposed to oxidizing gases (such as NO_3_), the resistance of the materials will increase. In contrast to n-type semiconductors, the resistance of p-type semiconductors is higher when exposed to reducing gas and decreases when exposed to oxidizing gas. At present, the most studied metal oxide materials for gas sensing are SnO_2_, ZnO, TiO_2_, CuO, WO_3_ and so on [57,104,105,106,107].

### 5.1. SnO_2_-Based Gas Sensors

SnO_2_ is a kind of direct band gap wide band gap n-type semiconductor (band gap ~ 3.6 eV), whose carriers are free electrons. The interaction with the reducing gas will increase the electrical conductivity. However, the oxidized gas will consume the sensing layer of charged electrons, resulting in a decrease in electrical conductivity [108]. SnO_2_ nanomaterials are widely used in the field of gas sensing because of their simple preparation, low cost, easy control of morphology, and microstructure, good thermal/chemical stability, shallow donor energy level (0.03–0.15 eV), potential barrier of oxygen adsorption on the surface is 0.3–0.6 eV, oxygen vacancy and excellent gas-sensing properties [109,110,111]. Thanks to its high sensitivity to different gases, SnO_2_ sensor can detect low concentration gases, but its selectivity is low.

In order to improve the sensitivity, stability, and selectivity of SnO_2_-based gas sensors and reduce the working temperature, researchers modified SnO_2_ materials by a variety of methods. One method is to control the morphology and size of SnO_2_ materials to prepare zero-dimensional (0D), one-dimensional (1D), 2D, three-dimensional (3D) and porous hollow SnO_2_ nanomaterials for the detection of various gases [112,113,114,115,116,117]. For example, Zhang et al. successfully synthesized leaf-like SnO_2_ hierarchical architectures by using a simple template-free hydrothermal synthesis method. The sensor based on this unique leaf-like SnO_2_ hierarchical structure had a high response and good selectivity to NO_2_ at low operating temperature [118]. Feng et al. synthesized mesoporous SnO_2_ nanomaterials with different pore sizes (4.1, 6.1, 8.0 nm) by carbon-assisted synthesis. The gas sensing properties of the three materials showed high sensitivity and ideal response recovery time to ethanol gas, and the detection limit was as low as ppb [119].

Using doping modification technology, SnO_2_ nanomaterials are used as the matrix materials of gas sensors, which are modified by doping precious metals (such as Pt, Pd and Au) or other metal ions (such as Ni, Fe and Cu). It is another important means to improve the gas sensing properties of SnO_2_ to CO, CH_4_, NO_2_ and other gases. For example, Dong et al. prepared SnO_2_ nanofibers and Pt-doped SnO_2_ nanofibers by electrospinning, which were used to test the sensitivity to H_2_S. It was found that the response of Pt-doped SnO_2_ nanofibers to H_2_S gas was significantly improved. The response of 0.08 wt% Pt-doped SnO_2_ nanofibers to 4–20 ppm H_2_S was 25.9–40.6 times higher than that of pure SnO_2_ nanofibers [120]. Chen et al. prepared Pd-doped SnO_2_ nanoparticles by the coprecipitation method. Compared with pure SnO_2_ nanoparticles, the response characteristics of SnO_2_ to CO were significantly improved [121]. Lee et al. used Pd nanoparticles to modify the surface of SnO_2_ nanorod thin films, and studied their sensing properties for H_2_ and ethanol gas [122]. It was found that compared with the undoped samples, the responsiveness of Pd-doped SnO_2_ nanorod thin films to 1000 ppm H_2_ and ethanol at 300 °C was increased by 6 and 2.5 times, respectively. They assumed that the improved gas sensing properties are due to the formation of the electron depletion layer and the enhanced catalytic dissociation of molecular adsorbates on the surface of Pd nanoparticles. Shen et al. also studied the gas-sensing properties of SnO_2_ by Pd doping [123]. SnO_2_ nanowires with a tetragonal structure were synthesized by thermal evaporation. The morphology, crystal structure, and H_2_ gas-sensing properties of undoped and Pd-doped SnO_2_ nanowires were studied. It was found that with the increase of Pd doping concentration, the working temperature decreased and the response of the sensor to H_2_ increased. Similarly, doping Au into SnO_2_ thin films can change the morphology of SnO_2_ thin films, reduce the grain size of SnO_2_ thin films, decrease the working temperature of the sensor, and improve the sensitivity and selectivity of SnO_2_ to reducing gases such as CO [124]. Zhao et al. carried out Cu doping on SnO_2_ nanowires. Compared with undoped SnO_2_ nanoscale arrays, the sensitivity and selectivity of the sensor to SO_2_ in a dry environment were improved significantly [125].

In addition, the researchers synthesized composite nanomaterials containing two different energy band structure materials to form heterostructures to improve the gas sensing performance of SnO_2_-based gas sensors. For example, Chen et al. prepared Fe_2_O_3_@SnO_2_ composite nanorods with multi-stage structure by a two-step hydrothermal method and found that the composite structure has good selectivity for ethanol [126]. Xue et al., using SnO_2_ nanorods synthesized by hydrothermal method as carriers, obtained SnO_2_ composite nanorods loaded with CuO nanoparticles by ultrasonic and subsequent calcination in Cu (NO_3_)_2_ solution. The gas sensing properties of the materials for the detection of H_2_S were studied. It is found that the sensitivity of the sensor to 10 ppm H_2_S can reach 9.4 × 10^6^ at 60 °C [127]. The ultra-high sensitivity of the composite is attributed to the p-n junction formed between CuO and SnO_2_. In the air, the formation of heterojunction increases the height of the energy barrier, hinders the flow of electrons, resulting in an increase in the resistance of the material. When the material is in contact with H_2_S and reacts, it can form CuS, which is similar to metal conductivity, which greatly enhances the electrical conductivity of the material. Fu et al. prepared NiO-modified SnO_2_ nanoparticles, which increased the thickness of the electron depletion layer on the surface of SnO_2_ through the formation of p-n heterojunction in air. While in the SO_2_ atmosphere, NiO reacted with SO_2_ to form NiS, which promoted the release of electrons from the surface adsorbed O^−^ to SnO_2_, thus enhancing the response of the device to SO_2_ gas and improving the gas sensitivity of SnO_2_ materials to SO_2_ [128].

### 5.2. ZnO-Based Gas Sensors

**Figure 8:**
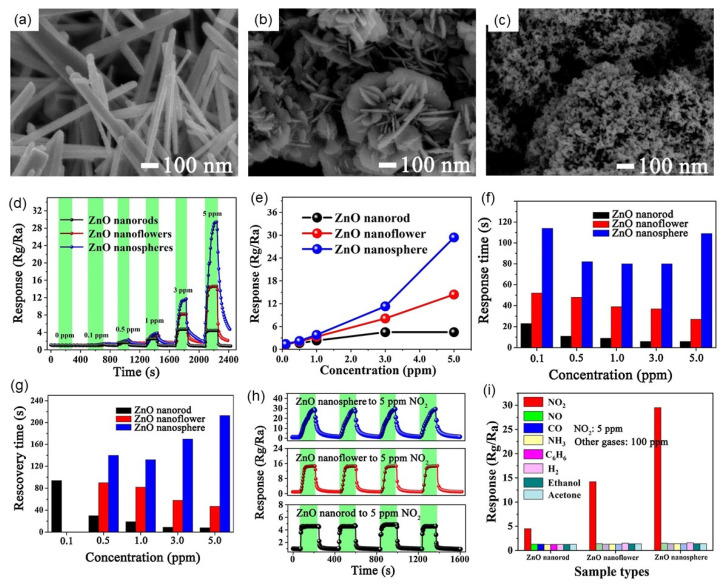
SEM images of (**a**) ZnO nanorods, (**b**) ZnO nanoflowers and (**c**) ZnO nanospheres. (**d**) Dynamic response curves with time of three different ZnO nanostructures; (**e**) the response curves with NO_2_ concentration of three different ZnO nanostructures; (**f**,**g**) the response and recovery time of three different ZnO nanostructures; (**h**) the repeatability of three different ZnO nanostructures to 5 ppm NO_2_; (**i**) the selectivity of three different ZnO nanostructures to other harmful gases. Reprinted with permission from Ref. [130]. Copyright 2021 Elsevier.

ZnO is an n-type metal oxide semiconductor material with a wide band gap (3.3 eV). ZnO is widely used in the field of gas sensors because of its good chemical stability and low resistivity. Yuliarto et al. successfully synthesized ZnO nanorod thin films on Al_2_O_3_ substrates by chemical bath deposition (CBD) [129]. ZnO thin films with different thicknesses were prepared by different times of CBD processes. By optimizing the thickness of ZnO thin films, the response performance of ZnO-based gas sensors to SO_2_ was improved. The gas sensing response of the ZnO film of two CBD to 70 ppm SO_2_ at 300 °C is 93%, which is 15% higher than that of the ZnO film of one CBD. At different operating temperatures, the response of ZnO nanorods prepared by two CBD deposition is 20–40% higher than that of ZnO nanorods deposited by one CBD deposition. Wang et al. successfully prepared three kinds of ZnO nanostructures (nanorods, flowers, and spheres) with different morphologies by a simple hydrothermal and water-bath method, and studied their sensing properties of NO_2_ at room temperature under UV (365 nm LED) excitation, as shown in Figure 8 [130]. It was found that ZnO nanospheres have the highest response (29.4) to 5 ppm NO_2_ (Figure 8d,e), which was mainly due to the largest specific surface area and the largest number of oxygen ions adsorbed on the surface of ZnO nanospheres. However, due to the high crystallinity, few surface defects and unidirectional electron transfer path, the response speed and recovery speed of ZnO nanorods are the fastest (9 s and 18 s, respectively) (Figure 8f,g). For ZnO nanoflowers, the gas sensing response, response and recovery rate are between ZnO nanorods and ZnO nanospheres. All three kinds of ZnO have good selectivity and repeatability for NO_2_ (Figure 8h,i). The good selectivity of ZnO to NO_2_ is attributed to the following two points: (1) NO_2_ molecule has an unpaired electron, which is beneficial to its chemisorption on ZnO surface; (2) NO_2_ molecule has the smallest bond energy, which is about 312.7 kJ/mol. The smaller the bond energy is, the more favorable the sensing reaction is, especially for the sensors working at room temperature.

Similar to the SnO_2_-based gas sensor, researchers changed the cell parameters of the original ZnO by element doping, making it produce lattice deformation, cause the surface defects of the gas sensing materials, and increase the surface active sites, so as to improve the gas sensing properties of the sensitive materials [131,132]. For example, Chaitra et al. prepared Al-doped ZnO thin films by the sol–gel method and spin-coating technique [133]. It was found that 2 at.% Al-doped ZnO thin films have the highest sensitivity to 3 ppm SO_2_ gas at 300 °C, which was lower than the threshold limit. Kolhe et al. prepared Al-doped ZnO thin films by chemical spray pyrolysis [134]. It was found that the doping of Al in ZnO led to the fracture of thin nanofilms, resulting in more active sites. Al doping also leads to the increase of oxygen vacancy-related defects and the change of crystal size due to the difference of ion radius between Al^3+^ and Zn^2+^ ions. The doped sensor has enhanced sensing characteristics, which also leads to the decrease of the optimal operating temperature. Xiang et al. used the photochemical method to embed Ag nanoparticles into ZnO nanorods and studied their gas-sensing properties [135]. It was found that Ag nanoparticles embedded on the surface of ZnO nanorods could improve the performance of the sensor. The response of ZnO nanorods to 50 ppm ethanol was almost three times that of pure ZnO nanorods, and had long-term stability. After 100 days of exposure to ethanol in 30 ppm, the response of the sensor had no obvious degradation.

**Figure 9:**
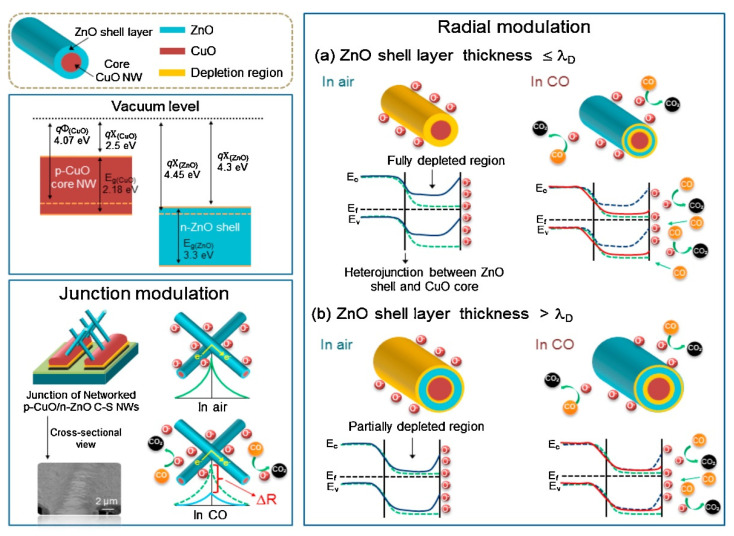
Schematic of the reducing gas sensing mechanism in the CuO–ZnO C–S NWs. Ec and EF indicate the conduction band energy and Fermi energy level, respectively, in cases of ZnO shell layers (**a**) thinner and (**b**) thicker than ZnO’s Debye length. Reprinted with permission from Ref. [136]. Copyright 2016 Elsevier.

The heterostructure is an important means to improve the gas sensing properties of semiconductor oxides, which usually includes two kinds of semiconductor oxides with different Fermi levels. When two kinds of semiconductor oxides come into contact with each other, the free electrons will change from the oxidation stream with a higher Fermi level to the oxide with a lower Fermi level. Compared with the single semiconductor oxide, the electron transfer efficiency of the two semiconductor oxides is higher, and a thicker electron depletion layer and higher resistance can be formed at the contact interface. Therefore, the introduction of heterojunction can effectively improve the performance of the semiconductor gas sensor. For example, Kim et al. synthesized p-n CuO/ZnO core–shell nanowires by thermal oxidation and atomic layer deposition, and studied their sensing properties to reduce gas by controlling the thickness of the ZnO shell [136]. When the shell thickness is less than or equal to Debye wavelength (λ_D_), a complete electron depletion layer will be formed. When exposed to the reducing gas (CO), the desorption of surface oxygen releases electrons back into the conduction band of the shell, returning the conduction band to its original state and significantly improving the conductivity (Figure 9a). When the thickness of the shell is higher than λ_D_, only part of the electron loss will be caused. When reducing gases are introduced, they are adsorbed on the partially depleted shell, and the resistance changes only slightly, as shown in Figure 9b. Therefore, for p-n heterostructure nanowires, controlling the shell thickness plays an important role in improving the performance of gas sensors. Zhou et al. prepared NiO/ZnO nanowires by one-step hydrothermal method and tested their gas sensing properties to SO_2_ [137]. It was found that at the optimum operating temperature of 240 °C, the response of NiO/ZnO nanowires to 50 ppm SO_2_ was 28.57, and the gas detection range was 5–800 ppm. The response time, response time and recovery time of the prepared NiO-ZnO nanowires gas sensor to 20 ppm SO_2_ gas were 16.25, 52, and 41 s, respectively.

### 5.3. CuO-Based Gas Sensors

**Figure 10:**
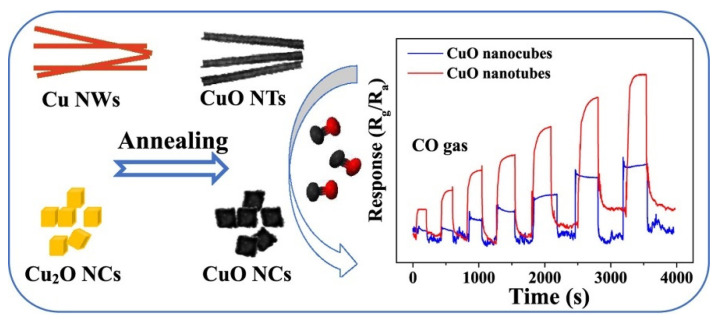
The preparation of CuO NTs and CuO NCs and CO gas-sensing behaviors of CuO NTs and CuO NCs at the operation temperature of 175 °C with different CO concentrations (50–1000 ppm). Reprinted with permission from Ref. [142]. Copyright 2018 Elsevier.

CuO is a typical p-type semiconductor oxide material with a band gap of 1.2–1.9 eV. Because of its good electrical properties, chemical stability, catalytic activity and other physical and chemical properties, CuO has been widely studied in the fields of catalysis, optoelectronic devices, gas sensors and so on. CuO can respond to reducing gases at lower operating temperatures, which attracts researchers to prepare different morphologies of CuO, doped CuO and heterostructure CuO for gas sensors to study their gas sensing properties. For example, Li et al. prepared porous CuO nanosheets on alumina tubes by hydrothermal method, which were used to make gas sensors to detect H_2_S [138]. It was found that when the concentration of H_2_S is as low as 10 ppb, the response sensitivity of the sensor was 1.25, and the response and recovery time were 234 and 76 s, respectively. Navale et al. synthesized CuO thin films that composed of CuO nanocubes on quartz substrates by simple and catalyst-free thermal evaporation technique, and studied their gas sensing properties [139]. It was found that CuO thin films have strong selectivity for NO_2_ gas, and the response speed and recovery time are fast. At 150 °C, the maximum response value of CuO sensor film to NO_2_ of 100 ppm was 76, the detection limit was 1 ppm, and the response time was only 6 s, but the recovery time was 1200 s. Huang et al. prepared CuO hollow microspheres by precipitation annealing at 270 °C using CuSO_4_, Na_2_CO_3_, and cetyltrimethyl ammonium bromide (CTAB) as raw materials [140]. The CuO hollow microspheres showed good gas sensitivity to ethanol. The response sensitivity to ethanol at 250 °C was 5.6 and the response and recovery times were 17.0 and 11.9 s, respectively. Hu et al. fabricated CuO nanoneedle arrays directly on commercial ceramic tubes by magnetron sputtering, wet chemical etching and annealing, which have good selectivity, reproducibility and long-term stability for low concentration H_2_S (10 ppm) [141]. For metal oxide semiconductors, high specific surface area and exposed crystal plane are two key factors that determine their gas sensing properties. In order to study the effect of surface structure on gas sensing properties, Huo et al. obtained CuO nanotubes on (111) exposed surfaces and CuO nanocubes on (110) exposed surfaces in Cu nanowires and Cu_2_O nanocubes, respectively, which were used to detect the gas sensing properties of CO gas, as shown in Figure 10 [142]. The results indicated that compared with CuO nanocubes, CuO nanotubes have lower optimal operating temperatures and higher sensitivity for CO gas detection.

Although pure CuO as a sensitive material can be used to detect a variety of toxic and harmful gases. However, it still faces some problems in practical applications, such as low sensitivity, high working temperature, poor selectivity, long response/recovery time and so on. For this reason, researchers use doping, recombination and other methods to improve the gas sensing performance of CuO-based gas sensors, and achieved some remarkable results. The doping of precious metals or rare earth elements can greatly increase the active sites of CuO gas-sensing reaction, which is beneficial to the adsorption of gas molecules on the sensitive material surface, and most of the dopants have strong catalytic activity, which can further enhance the gas sensing reaction. For example, Hu et al. prepared CuO nanoflowers with different Pd-doping concentrations by simple water-bath heating method [143]. Compared with pure CuO, the specific surface area of CuO nanoflowers with a size of about 400 nm prepared when the mass fraction of Pd was 1.25% increased by 1.8 times, and the response (Rg/Ra) to 50 ppm H_2_S at 80 °C was 123.4, which was 7.9 times that of pure CuO. In addition, the gas sensor has good stability and repeatability. Tang et al. prepared Pt-doped CuO nanoflowers by the same method, which significantly improved the gas sensing performance of the sensor to H_2_S gas [144]. When the amount of Pt doping was 1.25 wt.%, the response of the sensor to 10 ppm H_2_S at 40 °C was 135.1, which was 13.1 times that of pure CuO. The researchers also selected other metal elements to dope CuO, and achieved excellent results. For example, Mnethu et al. reported a highly sensitive and selective Zn-doped CuO nano-chip-based sensor [145]. At 150 °C, the response of 0.1 at.% Zn-doped CuO samples to 100 ppm xylene gas was 53. Bhuvaneshwari et al. reported a Cr-doped CuO nanoboat, which significantly improves the sensing performance of NH_3_ in the concentration range of 100–600 ppm at room temperature [146]. The gas sensing test results show that the sensitivity of CuO nanospheres doped with atomic fraction 6% Cr to NH_3_ at room temperature was 2.5 times higher than that of undoped nanospheres. The enhanced gas sensing performance is attributed to the increase of oxygen vacancy caused by chromium doping, which makes the nanospheres absorb more surface oxygen, and chromium doping also reduces the activation energy of the sensor at low temperatures. Al-doped CuO [147], In-doped CuO [148], and Ag-doped CuO [149] also showed excellent gas sensing properties for target gases.

The composite gas sensor can integrate the unique properties of the material and improve the performance of the sensor through complementary enhancement. Researchers have designed a variety of CuO-based composite gas sensors to improve the selectivity of target gases, enhance gas sensing properties, shorten response/recovery time and reduce the optimal operating temperature, especially semiconductor oxides with heterostructures. For example, Sui et al. used the template-free hydrothermal method to grow multi-layer heterogeneous CuO/NiO nanowires on ceramic tubes for the detection of H_2_S gas [150]. The CuO/NiO-based sensor has a wide linear range in the 50~1000 ppb range and has good repeatability, selectivity and long-term stability. At 133 °C, the 2.84 at.% CuO modified NiO showed good sensing properties, and the response to 5 ppm H_2_S was 36.9, which was 5.6 times higher than that of NiO. The detection limit of H_2_S is further reduced from 1 ppb of pure NiO sensor to 0.5 ppb. Park et al. synthesized SnO_2_-CuO hollow nanofibers by electrospinning and thermal processing, which can be used in the field of H_2_S gas sensing [151]. The electrospun nanofiber materials have the advantages of large surface area, high porosity and permeability to air or moisture, which is conducive to ionic diffusion and suitable for applications in gas sensors, lithium-ion batteries and wound healing [152,153]. SnO_2_-CuO nanotubes increase the specific surface area, decrease the working temperature and improve the sensing performance of H_2_S. At the working temperature of 200 °C, the sensitivity of hollow SnO_2_-CuO nanotubes to 5 ppm H_2_S was 1395 and the response time was 5.27 s. Liang et al. also prepared the heterostructure of In_2_O_3_ nanofibers supported on CuO by electrospinning and studied the sensing properties of H_2_S [154]. The gas sensor based on the heterostructure had a high sensitivity to 5 ppm H_2_S gas at 150 °C, which was 225 times higher than that based on pure In_2_O_3_, even at room temperature. The above research results show that the construction of heterojunction composites can effectively improve the sensitivity and selectivity of the gas sensor, reduce the working temperature, accelerate the response/recovery speed and prolong the life of the sensor.

### 5.4. Other Metal Oxide-Based Gas Sensors

WO_3_ is a kind of n-type wide band gap semiconductor oxide, which has the advantages of photoelectric conversion, electrochromism, photocatalysis, and gas sensitivity, so it is used in a variety of optoelectronic devices. WO_3_ is more likely to form oxygen defects and unsaturated coordination bonds. When WO_3_ is heated in the air, it is easy for O_2_ to seize e^−^ to form O^−^. The formed O^−^ is chemically adsorbed on the surface of WO_3_ and forms an electron depletion layer. When operating at a low temperature, it is easy to form O^2−^. when it is at a higher operating temperature, it is easy to form O^−^ and O^2−^ [155,156]. Therefore, WO_3_ is an effective gas sensing material, especially more sensitive to reducing gas. For example, Hu et al. synthesized WO_3_ nanorods with needle-shape via a hydrothermal mehod and subsequent calcination, which showed high performance for triethylamine gas sensing [157]. By comparison, it was found that WO_3_ nanosheet devices showed the highest response and the shortest response time to 1–10 ppm SO_2_. Li et al. proposed a method for the synthesis of WO_3_ particles assisted by ionic liquids. The hollow sphere structure composed of WO_3_ nanorods, nanoparticles and nanosheets was synthesized. Their gas sensing properties for various organic compounds (methanol, ethanol, isopropanol, ethyl acetate and toluene) were studied. It was found that it has remarkable sensitivity, low detection limit and fast response/recovery time [158]. Li et al. prepared SnO_2_-WO_3_ hollow nanospheres with a diameter of about 550 nm and a thickness of about 30 nm by hydrothermal method, and studied the temperature dependence of humidity sensors prepared at different relative humidity and temperature. It is found that compared with the original WO_3_ nanoparticles and SnO_2_ nanoparticles, SnO_2_-WO_3_ hollow nanospheres have excellent sensing properties [159].

α-Fe_2_O_3_ is also a typical n-type semiconductor with a narrow band gap (2.2 eV), low cost, high stability, high corrosion resistance, and non-toxicity, so it has attracted great attention as a gas sensing material [160,161]. Liang et al. successfully synthesized ultrafine and highly monodisperse α-Fe_2_O_3_ nanoparticles with an average particle size of 3 nm by a simple reverse microemulsion method, which showed high sensitivity, high selectivity and good stability to acetone [162]. Shoorangiz et al. synthesized α-Fe_2_O_3_ nanoparticles by a sol–gel method and evaluated their gas-sensing properties for ethanol and other gases [163]. At the optimal sensing temperature of 150 °C, it has good selectivity to ethanol gas, and the response to 100 ppm ethanol gas was 14.5%. Qu et al. reported high-performance gas sensors based on MoO_3_ nanoribbons that were modified by Fe_2_O_3_ nanoparticles [164]. Compared with the original MoO_3_ nanoribbons, the reaction of p-xylene in the Fe_2_O_3_ nanoribbons modified by Fe_2_O_3_ nanoparticles increased by 2–4 times due to the formation of heterojunction between Fe_2_O_3_ and MoO_3_.

As a p-type single metal oxide semiconductor, Co_3_O_4_ is also used to test gas sensitivity. It has been found to have a good gas sensing response to H_2_S in some studies [91,165]. Among different types of metal oxides, p-type Co_3_O_4_ is also considered to be the best candidate for ethanol gas sensing. For example, Li et al. reported that Co_3_O_4_ nanotubes are sensitive to ethanol gas at room temperature, and Co_3_O_4_ sensors show excellent repeatability after more than 50 tests [166]. Sun et al. obtained monodisperse porous Co_3_O_4_ microspheres by solvothermal method and thermal decomposition [167]. The gas sensing properties of these Co_3_O_4_ microspheres were compared with those of commercial Co_3_O_4_ nanoparticles. These Co_3_O_4_ microspheres showed higher ethanol sensitivity and selectivity at relatively low temperatures. In addition, Zhang et al. synthesized three kinds of Co_3_O_4_ with different morphologies (cube, rod, and sheet) by a hydrothermal method, and studied their sensing properties to toluene [168]. It was found that the sensor based on the Co_3_O_4_ flake structure had better sensing performance for toluene than the other two sensors. At the working temperature of 180 °C, the fabricated sensor showed higher sensitivity, faster response and recovery speed, as well as better selectivity.

## 6. 2D Materials/Metal Oxide-Based Gas Sensors

As a class of important materials for various sensors, semiconducting metal oxides have been widely used in various redox gas sensing due to their high sensitivity, simple preparation, and low price. However, disadvantages such as high temperature and easy agglomeration can also be found. Especially the disadvantage of high working temperature makes it extremely demanding on the working conditions of the environment, which greatly affects the service life. 2D materials such as graphene have unique physical and chemical properties, which can generate gas-sensitive responses at room temperature and have good selectivity. 2D materials can not only provide active sites for catalysts and sensors, but also serve as flat building blocks for forming complex nanostructures. Using 2D materials as a matrix to support semiconducting metal oxides can reduce the agglomeration of metal oxides and expose more adsorption and reaction sites. Due to the large specific surface area and high porosity of 2D materials, the response sensitivity and selectivity of metal oxides to specific gases can be further improved, and the working temperature can be reduced. Meanwhile, the synergistic effect and heterostructure of layered 2D materials and metal oxides can not only bring out the greatest advantages of both components but also overcome their respective defects, thereby improving the comprehensiveness of gas sensing performance. Therefore, the combination of 2D materials and metal oxides has become an important research direction in the field of gas sensors.

### 6.1. Synthesis of 2D Materials/Metal Oxide Composites

The properties of a material have a great relationship with its morphology, structure, and its composition. Single-component nanomaterials are far from meeting the development and application needs of modern nanotechnology, while multi-component composite materials combine the characteristics of different materials show better performance than their single components. It is of great significance in developing new materials, studying novel properties of materials, and constructing functional gas sensors. The preparation methods of 2D materials/metal oxide composites mainly include hydrothermal synthesis, microwave-assisted synthesis, self-assembly, chemical reduction method, and others. In this section, we would like to present a brief introduction to these potential synthesis methods for 2D materials/metal oxide composites.

Hydrothermal synthesis is a common method for the preparation of 2D materials/metal oxide nanocomposites, which has the advantages of simple operation, mild conditions, and low cost. Usually, the precursor solution is put into a high-pressure reactor, hydrothermally reacted under high temperature and high pressure, and then the composite material is prepared by post-processing methods such as separation, washing, and drying. Many 2D materials/metal oxide nanocomposites have been synthesized and used for the fabrication of gas sensors. For example, Chen et al. [169] prepared a core–shell structure composed of TiO_2_ nanoribbons and Sn_3_O_4_ nanosheets by a two-step hydrothermal reaction. Sn_3_O_4_ nanosheets were uniformly immobilized onto the surface of porous TiO_2_ nanoribbons. It was found that the structural morphology of the products in the hydrothermal process is affected by the reaction time. Chen et al. [170] investigated the effect of hydrothermal reaction temperature on the morphology of oxide materials. At a relatively high temperature, they obtained SnO_2_-decorated TiO_2_ nanoribbons. Wang et al. [171] obtained a composite structure based on SnO_2_ nanoparticles and TiO_2_ nanoribbons by controlling the precursor solution. Precise control of the hydrothermal synthesis conditions is a key factor for the preparation of high-quality and diversely shaped metal oxide nanostructures. In addition to metal oxide-based 2D composites, the composites of metal oxides and other 2D materials such as graphene have also been prepared by hydrothermal methods. For example, Chen et al. [172] prepared Co_3_O_4_/rGO composites by the hydrothermal method, and studied their gas-sensing properties to NO2 and methanol at room temperature. Chen and co-workers [173] synthesized SnO_2_ nanorods/rGO composite nanostructures by hydrothermal method and investigated their NH_3_ sensing properties. Liu et al. [174] fabricated a layered flower-like In_2_O_3_/rGO composites by a one-step hydrothermal method. The synthesized materials were further utilized for the fabrication of gas sensors, which exhibited improved sensing performance for 1 ppm NO_2_ at room temperature compared with pure In_2_O_3_-based gas sensors. The hydrothermal synthesis usually reacts at high temperatures (>150 °C). When the metal oxide is coupled with 2D materials for gas sensing, the operation temperature will be decreased, which is lower than the materials preparation temperature. Thus, the thermal stability and applicability of the heterostructures for gas sensors can be improved.

The microwave-assisted synthesis of materials uses microwaves to provide energy for the reaction, and it is different from the traditional heating method. It uses microwaves to make the reactants generate heat by themselves to promote the reaction. It has the characteristics of uniform heating and high heating efficiency. For example, Pienutsa et al. synthesized SnO_2_ decorated RGO and further used the created SnO_2_-RGO composite for the real-time monitoring of ethanol vapor [175]. In another similar study, Kim et al. [176] obtained SnO_2_/graphene heterostructured composites by microwave-treating graphene/SnO_2_ nanocomposites, in which graphene-enhanced efficient transmission of microwave energy and facilitated the evaporation and redeposition of SnO_x_ nanoparticles.

Chemical reduction has been often used to synthesize composite nanostructures of RGO and metal oxides. Usually, a metal salt solution is used as a precursor to be mixed with a graphene oxide dispersion solution, and a chemical reducing agent is used to reduce it in one step to obtain RGO. This method usually relies on a microwave, hydrothermal reaction, and other sources to provide energy. For example, Russo et al. [177] synthesized SnO_2_/rGO composites using SnCl_4_ and GO as precursors. Under the irradiation of microwave, GO and SnCl_4_ were reduced to form SnO_2_/rGO composites, which were further reacted with H_2_PtCl_6_ to form Pt-SnO_2_/rGO composites. The created composites exhibited high sensitivity to hydrogen at room temperature.

**Figure 11:**
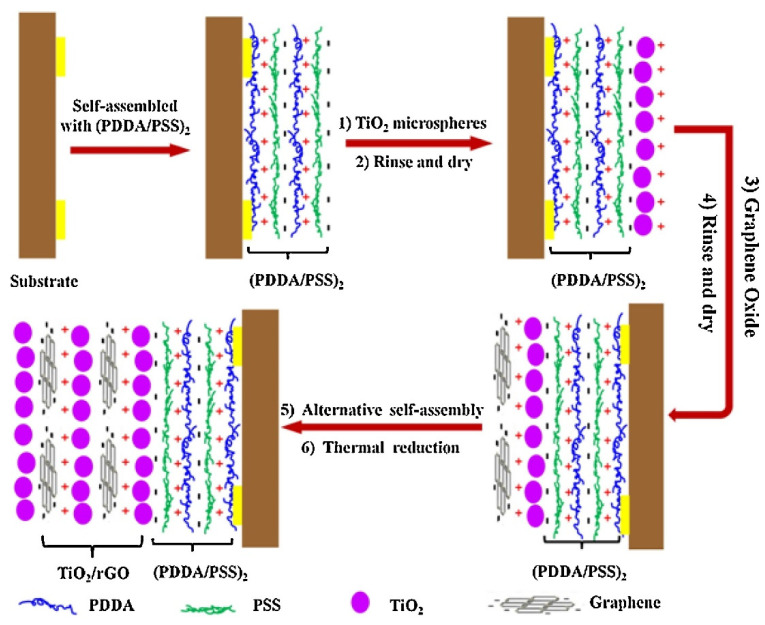
Fabrication illustration of TiO_2_/rGO multilayer hybrid film by layer-by-layer self-assembly. Reprinted with permission from Ref. [178]. Copyright 2017 Elsevier.

Besides the above-introduced methods, other techniques such as self-assembly can also be utilized for the synthesis of 2D materials/metal oxide composites. For instance, Zhang et al. fabricated rGO/TiO_2_ multilayer composite films using a layer-by-layer self-assembly process, and the fabrication process is shown in Figure 11 [178]. The rGO/TiO_2_ multilayer composite films were fabricated by alternately depositing TiO_2_ nanospheres and GO via the layer-by-layer self-assembly technique to form nanostructures, and then thermally reducing GO to rGO. Since p-type rGO and n-type TiO_2_ form a p-n heterojunction at the interface, the depletion layer generated by the built-in electric field will be beneficial to control the carrier transport process inside the material. It is clear that SO_2_ acts as an electron donor, which increases the electron concentration of the composite material, resulting in a decrease in device resistance. The device could be used to detect SO_2_ gas at as low as 1 ppb at room temperature with good selectivity and stability. The response of the fabricated gas sensor to 1 ppm SO_2_ was 10.08%, and the response and recovery times were 95 and 128 s, respectively.

### 6.2. Graphene/Metal Oxide Composite-Based Gas Sensors

Metal oxide-based gas sensors have the advantages of low production cost, good stability, wide application range, and easy integration with portable devices, and have been widely used in the measurement and monitoring of toxic and harmful gases [128,130,148,163]. However, since metal oxide-based sensors are limited by the required high operating temperature, they often bring additional energy consumption. In order to reduce the temperature for gas detection, composite materials are used as gas sensing materials. Taking advantage of the excellent gas sensing properties of metal oxides and the unique electrical, mechanical and thermodynamic properties of graphene, the formed graphene/metal oxide composites revealed high potential in the field of gas sensing.

Compared with traditional semiconductor gas sensing materials, graphene/metal oxide composite materials combine the advantages of the two materials and can produce synergistic effects for gas sensing, often with higher sensitivity, faster response/recovery speed, and lower noise signal. For example, Li et al. [179] prepared the rGO/ZnO hollow spheres by a one-step solvothermal method, in which the ZnO hollow spheres were uniformly immobilized on the surface of rGO nanosheets. When used as a material for the gas sensor to detect NO_2_, the composite material exhibited fast response and high sensitivity to NO_2_ at room temperature. In another case, Liu et al. synthesized rGO/ZnO composites by a redox method, and the prepared gas sensor had a response value of 25.6% to 5 ppm NO_2_ with a response time of 165 s and a recovery time of 499 s [180]. Sun et al. used PVP to assist the synthesis of the composite material rGO/ZnO nanowires. The gas-sensing performance test indicated that the rGO/ZnO composite material can respond to 500 ppb NH_3_ at room temperature, which is helpful for achieving the ultra-sensitive and high-accuracy detection of harmful gases [181]. Wang et al. used a one-step hydrothermal method to synthesize rGO/CuO/ZnO ternary composites to form nanoscale p-n junctions on rGO substrates. The gas sensors prepared by using the created materials showed excellent response characteristics and good selectivity to acetone, which was almost 1.5 and 2.0 times higher than those of CuO/ZnO and rGO/ZnO-based gas sensors, respectively [182]. It has become a research hotspot in the field of gas sensing to improve the performance of gas sensing materials by combining metal oxide semiconductor materials with 2D graphene materials.

Wang et al. successfully assembled SnO_2_ onto the surface of GO, and studied the gas-sensing properties of formaldehyde. It was found that SnO_2_ can be assembled on the surface of GO in a large area, and has good gas-sensing response properties to formaldehyde [183]. Wang et al. synthesized SnO_2_ nanoparticles onto RGO through a hydrothermal reduction to form SnO_2_-RGO composites, which exhibited promising application for room-temperature gas sensing of NO_2_ [184]. Yin et al. [185] synthesized SnO_2_/rGO nanocomposites with the SnO_2_ particle sizes of 3–5 nm uniformly immobilized on rGO nanosheets through a heteronuclear growth process by a simple redox reaction under microwave irradiation. The SnO_2_/rGO nanostructure on the surface has a sesame cake-like layered structure and an ultra-high specific surface area of 2110.9 m^2^·g^−1^. Compared with SnO_2_ nanocrystals (5–10 nm), the designed SnO_2_/rGO nanostructures have stronger gas-sensing behavior due to the unique hierarchical structure, high specific surface area, and synergistic effect of SnO_2_ nanoparticles and rGO nanosheets. At the optimal operating temperature of 100 °C, the SnO_2_/rGO-based gas sensor has a sensitivity as high as 78 and a response time as short as 7 s when exposed to 10 ppm H_2_S. In a similar study, Kim et al. [176] also synthesized a graphene/SnO_2_ composite material by the microwave-assisted method, and then sprayed the material onto SiO_2_ substrate to fabricate a NO_2_ gas sensor. At the optimal operating temperature of 150 °C, the response value of 1 ppm NO_2_ was 24.7. Its excellent gas-sensing response may be related to the homojunction between SnO_2_, the heterojunction between SnO_2_ and graphene, and the interstitial defects of Sn atoms in the SnO_2_ lattice.

Using the novel properties of SnO_2_, multi-walled carbon nanotubes (MWCNTs), and rGO, Tyagi et al. developed a hybrid nanocomposite sensor for efficient detection of SO_2_ gas [186]. The rGO-SnO_2_ and MWCNT-SnO_2_ composites were prepared by physical mixing and spin-coated onto the surface of Pt interdigital electrodes for SO_2_ gas detection. The sensing response of the bare SnO_2_ sensor to 500 ppm SO_2_ gas at 220 °C was 1.2. However, for the same concentration of SO_2_ gas, the enhanced sensing response of the MWCNT-SnO_2_ sensor was 5 at 60 °C, while the maximum sensing response of the rGO-SnO_2_ sensor was 22 at 60 °C. The enhanced SO_2_ gas sensing performance of these composites is mainly attributed to the p-n heterojunction formed at the interface between n-type SnO_2_ and p-type rGO or MWCNT.

**Figure 12:**
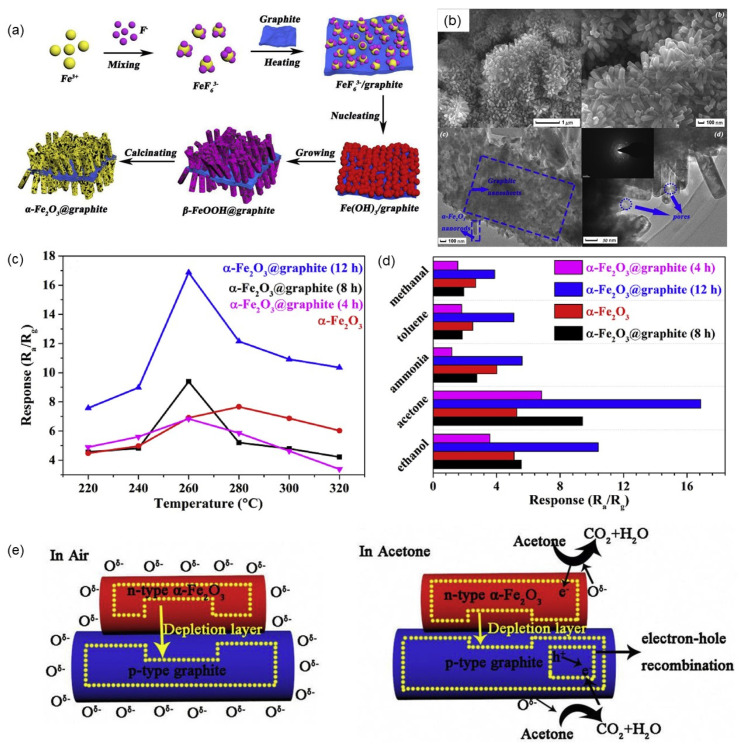
(**a**) Synthetic diagram of α-Fe_2_O_3_@graphite nanocomposites. (**b**) FE-SEM and TEM images of α-Fe_2_O_3_@graphite. (**c**) The responses to 50 ppm acetone of sensor based on α-Fe_2_O_3_ and α-Fe_2_O_3_@graphite (with different reaction times) operated at different operating temperatures. (**d**) The response of α-Fe_2_O_3_ and α-Fe_2_O_3_@graphite (with different reaction times) sensors to 50 ppm of different gases at 260 °C. (**e**) Reaction mechanism diagram of α-Fe_2_O_3_@graphite based gas sensor (O^δ−^ means O^−^, O^2−^ and O_2_^−^). Reprinted with permission from Ref. [187]. Copyright 2019 Elsevier.

Yu et al. [187] successfully synthesized α-Fe_2_O_3_@graphene nanocomposites using a simple low-temperature hydrolysis and calcination process, and fabricated the synthesized materials into gas sensors to detect different gases. Their prepared α-Fe_2_O_3_@graphite nanocomposites consist of porous α-Fe_2_O_3_ nanorods stably and orderly grown on graphitic nanosheets, as shown in Figure 12a,b. The length of α-Fe_2_O_3_ nanorods is related to the reaction time. When the reaction time was 12 h, the length reached a maximum value of about 200–300 nm, and the pore size was about 3.7 nm. Compared with pure α-Fe_2_O_3_, the α-Fe_2_O_3_@graphite nanocomposite-based sensor exhibited higher sensing performance for acetone. At the optimal temperature of 260 °C, the response of α-Fe_2_O_3_@graphite nanocomposites to 50 ppm acetone reaches a maximum value of 16.9, which was 2.2 times that of α-Fe_2_O_3_, as shown in Figure 12c. The high sensing performance was attributed to the porous structure, high specific surface area, and p-n heterostructure of α-Fe_2_O_3_@graphite nanocomposites and the high temperature stability of graphite. When α-Fe_2_O_3_ was recombined with graphite, due to the large gradient of the same carrier concentration, the electrons in α-Fe_2_O_3_ and the holes in graphite diffuse in opposite directions, so that a built-in electric field is formed between the interfaces, and the electrons in the depletion layer. The energy bands bend until the system reaches equilibrium at the Fermi level (E_F_), leading to the formation of a p-n heterojunction. Once the α-Fe_2_O_3_@graphite heterojunction sensor is exposed to acetone gas, the oxygen anions adsorbed on the sample surface undergo a redox reaction with acetone molecules and release electrons back into α-Fe_2_O_3_, resulting in a decrease in the resistance of the sensor. At the same time, acetone releases electrons to combine with holes in p-type graphite, resulting in a decrease in hole concentration. The reduction of holes in graphite leads to an increase in electrons and reduces the concentration gradient of the same carriers on both sides of the p-n heterojunction. Therefore, the diffusion of carriers was weakened and the barrier height of the depletion layer was reduced, which further reduced the resistance of the α-Fe_2_O_3_@graphite sensor, as shown in Figure 12e.

Co_3_O_4_, as a direct bandgap p-type metal oxide semiconductor material, has also received extensive attention in gas sensors and other fields due to its outstanding advantages such as strong corrosion resistance and non-toxicity. For instance, Zhou et al. [188] studied the performance of a Co_3_O_4_-based gas sensor and found that it can only work at temperatures over 200 °C. Zhang et al. [189] proposed that the rGO/Co_3_O_4_ nanocomposite-based sensor can realize the detection of NO_2_ gas at room temperature. Srirattanapibul et al. [190] prepared a Co_3_O_4_-modified rGO (rGO/Co_3_O_4_) nanocomposite-based gas sensor by a solvothermal method. Co_3_O_4_ nanoparticles were distributed on and between the rGO flakes, and their dosage changed the bandgap and gas sensing properties of rGO. Decorating rGO with Co_3_O_4_ nanoparticles promoted the formation of the Co-C bridges, which enable the exchange of charge carriers between Co_3_O_4_ nanoparticles and rGO flakes, thereby increasing the number of sites for gas reactions to occur and improving gas sensing performance. The as-prepared 25% rGO/Co_3_O_4_-based gas sensor has a sensitivity of 1.78% and a response time of 351 s towards 20 ppm NH_3_.

Many studies on the synthesis of the composites by combing graphene with other metal oxides have also been carried out. For example, Hao et al. [191] synthesized WO_3_/rGO porous nanocomposites using a simple hydrothermal and annealing process. The material-based gas sensor showed good sensitivity to NO_2_ and some volatile organic compounds. In another study, Ye et al. [192] fabricated uniform TiO_2_/rGO membranes with enhanced NH_3_ responsiveness by stepwise deposition of GO and TiO_2_ layers followed by simple thermal treatment. To make it more clear, here we summarize the gas sensing properties of the above graphene/metal oxide nanocomposite-based gas sensors, as shown in Table 1.

### 6.3. 2D TMD/Metal Oxide Composite-Based Gas Sensors

Compared with graphene with a zero-band gap, the electronic structure of 2D transition metal dichalcogenides (TMDs) almost spans the whole range of the electronic structure, showing richer physical properties and a good application potential in gas sensing. However, TMD nanosheets are easy to form a dense stack structure in the process of forming a conductive network, which is not conducive to the full contact between the thin sheet and gas molecules in the conductive network, so its sensitivity and response recovery speed at room temperature need to be improved. TMDs and other metal oxide semiconductor materials form p-n heterojunction as a new type of electronic device, which can improve its gas sensing performance. The unique characteristics of TMDs make the composites an ideal choice for high-performance sensing materials at low temperatures.

**Figure 13:**
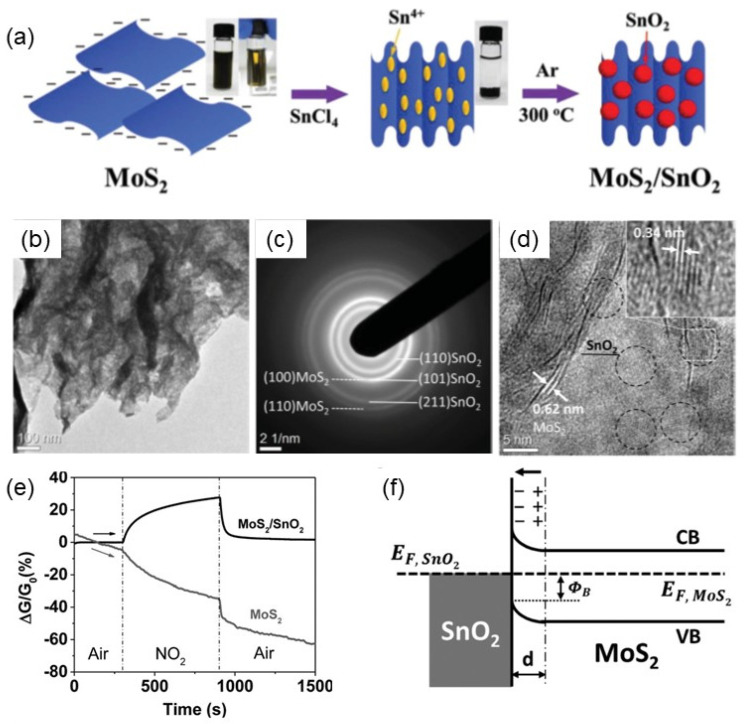
(**a**) Schematic illustration of the preparation process for MoS_2_/SnO_2_ nanohybrids. The inset photographs show the MoS_2_ suspension in water before and after adding the SnCl_4_ solution. (**b**) TEM images, (**c**) SAED pattern and (**d**) HRTEM images of the MoS_2_/SnO_2_ nanohybrids. The inset of (**d**) shows a typical SnO_2_ nanocrystal on the MoS_2_ surface. (**e**) The room temperature dynamic sensing response of MoS_2_ nanosheets with and without SnO_2_ NC decoration against 10 ppm NO_2_ in a dry air environment, indicating the SnO_2_ NCs significantly enhanced the stability of MoS_2_ in the dry air. (**f**) Band diagram of the MoS_2_/SnO_2_ nanohybrid. The EF, SnO_2_ and EF, MoS_2_ are Fermi levels of SnO_2_ and MoS_2_, respectively. The CB and VB are the conductance and valance band edges of MoS_2_, respectively. d is the thickness of the electron depletion zone, and ΦB is the Schottky barrier height. Reprinted with permission from Ref. [194]. Copyright 2015 John Wiley & Sons, Inc.

In the research of gas sensing applications, SnO_2_ has been the most widely used n-type semiconductor, so the combination of SnO_2_ and MoS_2_ is an effective way to improve the sensing performance of MoS_2_. For instance, Qiao et al. loaded MoS_2_ nanosheets onto SnO_2_ nanofibers by hydrothermal synthesis. Through the optimal regulation of MoS_2_/SnO_2_ heterostructure, they achieved high-performance detection of trimethylamine at 230 °C, showing excellent sensing selectivity and long-term stability [193]. In the field of room temperature gas sensors, Cui et al. reported a new hybrid material by decorating MoS_2_ nanosheets with SnO_2_ nanocrystals, which achieved high-performance sensing of NO_2_ at room temperature, and the loaded SnO_2_ nanoparticles could significantly enhance the stability of MoS_2_ nanosheets in the air (Figure 13) [194]. In the composite, SnO_2_ nanocrystals acted as a strong p-type dopant at the top of MoS_2_, resulting in the formation of p-type channels in MoS_2_ nanosheets. In terms of sensing mechanism, they believed that SnO_2_ nanocrystals may be the main gas adsorption center, while MoS_2_ acted as a conductive channel at room temperature. The close electrical contact between two different semiconductor materials led to the formation of charge transfer and charge depletion layer. Because the work function of SnO_2_ (5.7 eV) was larger than that of MoS_2_ (5.2 eV), electrons are transferred from MoS_2_ to SnO_2_, resulting in a depletion layer and a Schottky barrier (Figure 13f). These electron depletion regions can be connected to each other on the surface of MoS_2_ nanosheets and act as a passivation layer to prevent the interaction between oxygen and MoS_2_, thus enhancing the stability of MoS_2_ nanosheets in dry air. The fabricated gas sensor exhibited high sensitivity, excellent repeatability, and excellent selectivity to NO_2_ in the actual dry air environment, and the detection limit could reach 0.5 ppm.

In addition to SnO_2_, ZnO is another wide band gap n-type semiconductor for the fabrication of high-performance gas sensors. Yan et al. synthesized the ZnO/MoS_2_ composite structure by coating ZnO nanoparticles onto MoS_2_ nanosheets through a two-step hydrothermal method [195]. Among the composites, MoS_2_ is a multi-stage structure composed of nanosheets with a thickness of 5~10 nm, and the size of ZnO particles was about 8 nm. The response value of the composite-based gas sensor to 50 ppm ethanol reached 42.8 at the operating temperature of 260 °C Han et al. designed a MoS_2_/ZnO heterostructure on the MoS_2_ nanosheets obtained by liquid phase exfoliation by a wet chemical method, and achieved efficient detection of NO_2_ gas at room temperature [196]. After surface modification, ZnO nanoparticles had a good response to 5 ppm NO_2_, and the response value reached 3050%, which was 11 times higher than that of pure phase MoS_2_ nanoparticles. In addition, the recoverability of the heterostructure was improved to more than 90% without auxiliary means, and the sensor also had the characteristics of fast response speed (40 s), reliable long-term stability within 10 weeks, good selectivity, and a low detection concentration of 50 ppb. The enhanced sensing performance of MoS_2_/ZnO heterostructure can be attributed to the unique 2D/0D heterostructure, synergistic effect and the p-n heterojunction between ZnO nanoparticles and MoS_2_ nanosheets.

Besides, Zhao et al. reported the hydrothermal synthesis of MoS_2_-modified TiO_2_ nanotube composites and studied their gas sensing properties [76]. TiO_2_ nanotubes are filled and covered by 1–3 layers of flake MoS_2_ nanosheets. The formed MoS_2_-TiO_2_ composites revealed excellent sensing properties and high sensitivity to ethanol vapor at low operating temperatures, and their sensitivity was almost 11 times that of TiO_2_ nanotubes. The response to 100 ppm ethanol gas was ~14.2 and the optimum working temperature was as low as 150 °C. Zhang et al. prepared CuO/MoS_2_ heterostructure sensing films on the substrate by layer-by-layer self-assembly technique [197]. Compared with the pure phase CuO and MoS_2_, the formed CuO/MoS_2_ composite structure exhibited higher response, shorter response/recovery time, better repeatability, higher selectivity, and longer-term stable H_2_S detection performance. The excellent H_2_S sensing properties are mainly due to the existence of a large number of oxygen and sulfur vacancies in the composite structure of CuO nanorods and MoS_2_ nanosheets, which brings a large number of active sites for gas adsorption. In addition, the synergistic effect of binary nanostructures and the modulation of electron transfer by the formation of p-n heterojunction at the material interface between p-type CuO semiconductors and n-type MoS_2_ semiconductors promoted the performance of the composite structure. Ikram et al. prepared MoO_2_/MoS_2_ nanonetworks by controllable vulcanization and successfully applied them to the efficient detection of NO_2_ gas at room temperature [198]. The response value of the composite structure to 100 ppm NO_2_ gas was 19.4, and it had ultra-fast response speed and recovery speed, and the response and recovery time were 1.06 s and 22.9 s, respectively. The excellent gas-sensing performance of the sensor can be attributed to the synergistic effect between MoS_2_ nanosheets and MoO_2_ nanoparticles. The defects in the synthesis process provided more active sites for NO_2_ gas molecules, and the formation of p-n heterojunction accelerated the charge transfer between NO_2_ and gas molecules.

In addition to MoS_2_, other transition metal dichalcogenides and metal oxide composites have been also often used as sensitive materials for gas detection. For example, Qin et al. prepared 2D WS_2_ nanosheets/TiO_2_ quantum dots composites by chemical stripping method, which have been successfully used in room temperature NH_3_ sensing. The fabricated gas sensors exhibited a quicker sensing response to 250 ppm NH_3_, which was almost 17 times that of the original WS_2_ [199]. Gu et al. prepared SnO_2_/SnS_2_ heterojunction nanocomposites by the in situ high-temperature oxidizer SnS_2_, which significantly improved the response to NO_2_ and decreased the working temperature [200]. To make it more clear, the gas sensing properties of the transition metal dichalcogenides/metal oxide composite-based gas sensors are shown in Table 2.

### 6.4. Other 2D Material/Metal Oxide Composites-Based Gas Sensors

**Figure 14:**
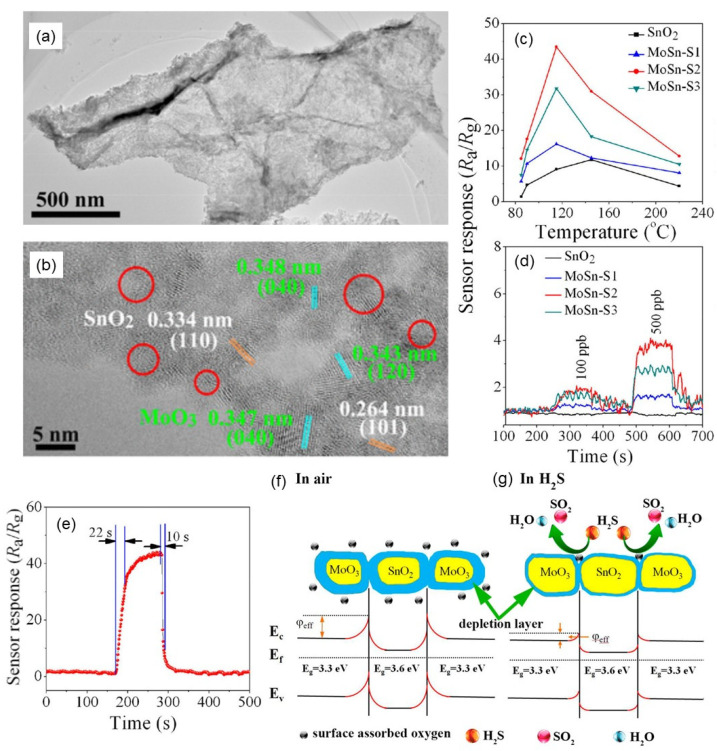
(**a**,**b**) Low-magnification TEM images and HRTEM images of MoSn-S_2_ nanoflakes. Red circles in (**b**) shows the lattice distortions at the interfaces between MoO_3_ and SnO_2_. (**c**) Sensor responses of as-prepared samples as a function of different operating temperatures to 10 ppm of H_2_S concentration. (**d**) Typical sensor responses of SnO_2_, MoSn-S1, MoSn-S_2_, and MoSn-S_3_ toward 100 and 500 ppb of H_2_S gas at optimal working temperature, and (**e**) response and recovery times curve of MoSn-S_2_ NFs to 10 ppm of H_2_S at 115 °C. (**f**,**g**) Diagram of energy band structure of MoSn−nanocomposites in (**f**) air and (**g**) H_2_S. Reprinted with permission from Ref. [203]. Copyright 2019 ACS.

As an important wide band gap and semiconductor gas sensing material with a special layered structure, MoO_3_ is easy to form metal phase MoS_2_ in the process of reacting with H_2_S, which makes MoO_3_ have unique response characteristics to H_2_S gas. However, single-phase MoO_3_ has some shortcomings, such as high working temperature and poor limit detection ability [201,202], so it needs to be compounded with other materials to improve its gas-sensing performance. For example, Gao et al. used graphene as a sacrificial template and prepared porous MoO_3_/SnO_2_ composite nanosheets with n-n heterostructure by hydrothermal method, and studied their gas sensing properties, as shown in Figure 14 [203]. The TEM characterizations indicated that the composite is a porous structure composed of MoO_3_ and SnO_2_ nanoparticles, and the lattice distortion at the interface indicates the existence of MoO_3_-SnO_2_ n-n heterojunction (Figure 14a,b). The gas-sensing performance test showed that compared with SnO_2_ nanosheets, the introduction of MoO_3_ and the formation of heterojunctions lead to the change of energy band structure and carrier separation transfer rate, and the optimum operating temperature of MoO_3_/SnO_2_ nanosheet sensor is lower and the sensitivity is higher. The detection limit of MoO_3_/SnO_2_ nanoparticles for H_2_S was as low as 100 ppb at 115 °C, and the response and recovery times were 22 s and 10 s, respectively (Figure 14c–e). The excellent gas-sensing performance of the MoO_3_/SnO_2_ composite nanosheet gas sensor was attributed to the following aspects.

Firstly, in the composites, the n-n heterojunction formed at the interface between MoO_3_ and SnO_2_, and the charge accumulation layer and charge consumption layer are formed on both sides of the heterojunction, respectively, thus forming an internal electric field and hindering the free transport of free electrons in the semiconductor. The sensing mechanism of the composite material is shown in Figure 14f,g. When the material is exposed to air, the electrons in the semiconductor MoO_3_ and SnO_2_ conduction bands adsorb the oxygen in the air to the surface of the semiconductor gas sensing material to form the adsorbed oxygen. This leads to the loss of electrons in the conduction bands of semiconductors MoO_3_ and SnO_2_ (Figure 14f). On the energy band diagram, the energy bands of semiconductors MoO_3_ and SnO_2_ bend upward, and the barrier height increases. In the atmosphere of H_2_S, when H_2_S reacts with the adsorbed oxygen on the surface of semiconductor sensitive materials, the electrons captured by adsorbed oxygen are released back into the semiconductor MoO_3_ and SnO_2_ conduction bands, which leads to a significant decrease in the barrier height at the n-n heterojunction and a significant decrease in the resistance of the sensor (Figure 14g). The electrical conductivity of composite semiconductor materials is inversely proportional to the barrier height, and the barrier height can effectively control the gas sensing response characteristics of sensitive materials with heterojunctions. Therefore, a large change of barrier height has a significant contribution to the H_2_S performance of the MoO_3_/SnO_2_ gas sensor. Secondly, the large specific surface area of MoO_3_/SnO_2_ composite nanosheets means that when exposed to H_2_S gas, the MoO_3_/SnO_2_ sensor with a large specific surface area can contact more H_2_S gas molecules and react with them, resulting in greater resistance change and higher sensitivity response. Thirdly, the composite has high porosity, and the larger pore volume can not only promote the adsorption of H_2_S gas molecules to the sensitive material surface more quickly, but also provide a shorter gas conduction path and promote the gas molecules to break away from the sensitive material surface and then achieve the ideal state of rapid response and rapid recovery.

Yin et al. [204] synthesized hierarchical Fe_2_O_3_/WO_3_ nanocomposites with ultra-high specific surface area composed of Fe_2_O_3_ nanoparticles and single-crystal WO_3_ nanosheets through microwave heating and in situ growth. The BET specific surface area of the sample that prepared with 5 wt.% Fe_2_O_3_/WO_3_ by this process was as high as 1207 m^2^·g^−1^, which was 5.9 times that of the corresponding WO_3_ nanosheets (203 m^2^·g^−1^). The significant enhancement of the specific surface area of the Fe_2_O_3_@WO_3_ samples was attributed to the hierarchical structure of the prepared composite materials, in which the monolayer and unconnected Fe_2_O_3_ nanoparticles are tightly anchored to the surface of the WO_3_ nanosheets, so that the inner surface or interface of the aggregated polycrystal is entirely the outer surface. The gas-sensing performance tests indicated that Fe_2_O_3_@WO_3_ nanocomposites exhibited high response and selectivity towards H_2_S at low operating temperatures due to the synergistic effect of the components of Fe_2_O_3_@WO_3_ nanocomposites and the hierarchical microstructure with ultra-high specific surface area. At 150 °C, the fabricated gas sensor showed a response to 10 ppm H_2_S of as high as 192, which was four times that of the WO_3_ nanosheet-based gas sensor.

## 7. Conclusions and Future Perspectives

In this paper, we summarize the research progress of gas sensors using 2D materials, metal oxides, and their composites as sensitive materials. The gas sensing mechanism, main factors affecting sensing performance, and the applications of various gas sensors are presented and discussed in detail. It can be concluded that the 2D material/metal oxide-based gas sensor can efficiently identify and detect toxic and harmful gases. Compared with pure metal oxide semiconductors, composite materials have higher carrier rates, larger high mechanical strength, and large specific surface area, and the synergistic effect of the two components can further enhance the gas sensing performance. The -OH, -O, and other functional groups on the surface of 2D materials not only provide chemical bonds to form composite metal oxide materials during the composite process of the material, but also give more active sites for the gas sensing process, thereby further improving the gas sensing performance. In addition, the composite materials can effectively reduce the working temperature.

Although nanomaterial-based gas sensors have made great progress in the past few decades, the operating temperature of metal oxides is too high, and the selectivity of 2D materials is still unsatisfactory. In the future, effective strategies such as building composite structures are highly needed to improve the selectivity, reduce the operating temperature, and improve the sensitivity and other properties. In addition, the research on the combination of metal oxides with 2D materials is still at an early stage, and its sensing mechanisms of the composite-based gas sensors should be further studied. Only when the mechanism and process are clear, the preparation and assembly of nanomaterial-based gas sensors can be achieved purposefully. In addition, it is necessary for researchers to develop new design strategies to further optimize metal oxide nanomaterials with 2D nanomaterials to make them more suitable for gas sensing. Finally, it is expected that facile assembly and fabrication processes will be developed to enable batch fabrication of gas sensors with high stability, selectivity, sensitivity, reproducibility, and quick response in the future.

## Figures and Tables

**Table 1:** Sensors based on MOS modified with graphene/GO/rGO gas sensing performances.

Sensor Materials	Analyte	Response	Working Temperature	Refs.
ZnO/rGO	NO_2_	17.4% (100 ppm)	RT	[179]
ZnO/rGO	NO_2_	25.6% (5 ppm)	RT	[180]
ZnO/rGO	NH_3_	7.2% (1 ppm)	RT	[181]
SnO_2_/GO	HCHO	32 (100 ppm)	120 °C	[183]
SnO_2_/rGO	H_2_S	78 (10 ppm)	100 °C	[185]
Graphite/SnO_2_	NO_2_	24.7 (1 ppm)	150 °C	[176]
rGO/SnO_2_	SO_2_	22 (500 ppm)	60 °C	[186]
α-Fe_2_O_3_@graphite	C_3_H_6_O	16.9 (50 ppm)	260 °C	[187]
rGO/Co_3_O_4_	NO_2_	26.8% (5 ppm)	RT	[189]
rGO/Co_3_O_4_	NH_3_	1.78% (20 ppm)	RT	[190]
WO_3_/rGO	NO_2_	4.3 (10 ppm)	90 °C	[191]
TiO_2_/rGO	NH_3_	0.62 (10 ppm)	RT	[192]

**Table 2:** Sensors based on MOS modified with TMDs gas sensing performances.

Sensor Materials	Analyte	Response	Working Temperature	Refs.
SnO_2_/MoS_2_	C_3_H_9_N	106.3 (200 ppm)	230 °C	[193]
SnO_2_/MoS_2_	NO_2_	28% (10 ppm)	RT	[194]
ZnO/MoS_2_	C_2_H_6_O	42.8 (50 ppm)	260 °C	[195]
ZnO/MoS_2_	NO_2_	3050% (5 ppm)	RT	[196]
MoS_2_/TiO_2_	C_2_H_6_O	14.2 (100 ppm)	150 °C	[76]
CuO/MoS_2_	H_2_S	61 (30 ppm)	RT	[197]
MoO_2_/MoS_2_	NO_2_	19.4 (100 ppm)	RT	[198]
TiO_2_ QDs/WS_2_	NH_3_	43.7% (250 ppm)	RT	[199]
SnO_2_/SnS_2_	NO_2_	5.3 (8 ppm)	80 °C	[200]

## Data Availability

The data presented in this study are available in insert article.
